# Reliable Hardware Sensor and Large Language Model Fusion for Intelligent Short-Term Market Risk Sensing and Prediction

**DOI:** 10.3390/s26175322

**Published:** 2026-08-22

**Authors:** Zijian Zhou, Nuo Wang, Shengzhe Xu, Surui Hua, Hanyang Wang, Yachi Liu, Manzhou Li

**Affiliations:** 1 Peking University, Beijing 100871, China; 2 Capital University of Economics and Business, Beijing 100070, China; 3 China Agricultural University, Beijing 100083, China

**Keywords:** large language models, hardware sensors, multimodal sensor fusion, asynchronous cross-modal alignment, sensor reliability modeling

## Abstract

Short-term financial risk in intelligent trading systems is reflected not only in prices, trading volumes, and textual sentiment but also in infrastructure operating states, including server workload, device power consumption, network latency, and packet loss rate. We propose HSF-LLMNet, a hardware sensor and large language model semantic fusion network for jointly modeling external information shocks and infrastructure responses. A large language model extracts event category, sentiment polarity, risk intensity, and semantic uncertainty from financial texts. Reliability-aware temporal modeling handles sensor missingness, drift, and abnormal noise, while asynchronous soft alignment, bidirectional cross-attention, and reliability-aware gated fusion integrate irregular textual events with continuous hardware signals. The model jointly predicts market direction, realized volatility, and three-level risk over the subsequent 30 min. Experiments were conducted on eight Chinese A-share indices: the SSE Composite Index (000001.SH), SSE 50 Index (000016.SH), CSI 300 Index (000300.SH), STAR 50 Index (000688.SH), CSI 500 Index (000905.SH), CSI 1000 Index (000852.SH), Shenzhen Component Index (399001.SZ), and ChiNext Index (399006.SZ). The common observation period for market, textual, and hardware data extended from 1 March 2024 to 30 June 2025. After data cleaning, timestamp matching, and multimodal temporal alignment, 169,208 aligned asset–time prediction windows were retained for the 30 min forecasting task. Realized volatility was defined as the square root of the sum of squared one-minute log returns over the future 30 min interval. The three-level risk label was constructed from future realized volatility, absolute 30 min return, and liquidity stress, with all thresholds estimated exclusively from the training portion of each fold. A sample was labeled high risk when at least two of the three indicators exceeded their 85th-percentile thresholds or when any indicator exceeded its 95th-percentile threshold. It was labeled medium risk when, after excluding high-risk samples, at least two indicators exceeded their 60th-percentile thresholds or any indicator exceeded its 85th-percentile threshold; all remaining samples were labeled low risk. Results showed that HSF-LLMNet achieved an accuracy of 78.62%, a precision of 78.14%, a recall of 77.83%, an F1-score of 77.98%, an area under the receiver operating characteristic curve of 84.91%, and a Matthews correlation coefficient of 57.36% for directional prediction. For realized-volatility regression, the MAE, RMSE, MAPE, and $R^{2}$ were 0.0089, 0.0135, 9.21%, and 0.812, respectively. For high-risk-event warning, the mean effective warning time, defined as the interval between the first valid alarm and the corresponding event, was 15.37 min; the false-alarm rate and missed-alarm rate were 6.82% and 8.14%, respectively. Ablation experiments showed performance reductions after removing semantic encoding, sensor-reliability estimation, asynchronous alignment, bidirectional cross-attention, gated fusion, or multi-task learning. These results indicate that textual events and infrastructure operating states provide complementary information for quantitative risk analytics and fintech applications.

## 1. Introduction

With the rapid development of intelligent trading systems, data centers, and automated trading platforms, modern markets have evolved into complex cyber–physical systems supported by large-scale computational infrastructures rather than being driven solely by investor behavior and market dynamics [1,2]. During major policy announcements, corporate disclosures, or extreme market conditions, market stress is reflected not only in prices, trading volumes, and textual sentiment but also in the operational status of trading infrastructures [3]. Hardware sensor signals, such as market request volume, server workload, network latency, power consumption, and edge resource utilization, can directly capture system pressure and trading congestion from the infrastructure perspective [4]. Incorporating these sensor observations into forecasting therefore provides a new paradigm that shifts market prediction from outcome analysis toward operational state perception [5]. Existing prediction methods mainly rely on historical prices, trading volumes, technical indicators, news, corporate disclosures, and social media sentiment [6]. Although these information sources have achieved promising performance, they still present several limitations [7]. Market variables such as prices and trading volumes are primarily outcome-oriented signals and often respond after market conditions have already changed, limiting their effectiveness for early risk warning [8]. Textual information is further affected by delayed dissemination, semantic ambiguity, noisy content, and sentiment manipulation, making it difficult to consistently capture real-time market dynamics [9]. Meanwhile, hardware monitoring data have traditionally been used only for infrastructure maintenance, fault diagnosis, and system security, while their potential value for forecasting has received little attention [10]. In practice, abnormal market events often trigger coordinated changes in both market behavior and infrastructure status. Ignoring these infrastructure-level signals limits the ability to characterize the dynamic transition from information shocks to system responses and ultimately to market volatility [11].

Recent advances in deep learning and large language models (LLMs) have improved text understanding, sentiment analysis, and market prediction [12]. Recurrent neural networks, Transformers, and graph neural networks have been widely applied to forecasting with prices, trading volumes, and textual information [13], while LLMs enable the extraction of event categories, sentiment polarity, risk intensity, and semantic uncertainty from news, corporate disclosures, and investor discussions [14]. Sensor-based temporal modeling has also been used for hardware-state monitoring, anomaly detection, and workload prediction [15]. However, existing multimodal studies mainly combine textual and market data [16,17], and it remains unclear whether infrastructure measurements provide predictive information beyond observable prices, trading volumes, and textual semantics.

Accordingly, the central research question of this study is whether trading-infrastructure sensor measurements provide incremental out-of-sample information for predicting market direction, realized volatility, and risk state over the subsequent 30 min after controlling for historical prices, returns, trading volumes, trading values, liquidity variables, and textual semantic information. This distinction is necessary because server utilization, power consumption, network throughput, and packet traffic may merely respond to contemporaneous trading activity. We therefore test whether the sensor contribution remains when market and textual variables are included, when sensor features are residualized with respect to contemporaneous trading volume, trading value, absolute return, and historical realized volatility, and when sensor observations from the final 5 or 10 min before the prediction origin are excluded. Performance gains that disappear under these controls are interpreted as proxy effects, whereas persistent gains indicate incremental leading information.

To address this research question, we propose HSF-LLMNet, a hardware sensor and large language model semantic fusion framework for short-term market prediction. The framework integrates semantic representations extracted from financial texts with reliability-aware temporal representations of infrastructure operating states. A cross-frequency temporal alignment module associates irregular textual events with continuous sensor sequences, while the hardware encoder handles missingness, drift, and abnormal observations. Bidirectional cross-attention and reliability-aware gated fusion are then used to model delayed and asymmetric interactions between semantic shocks and infrastructure responses. The proposed model is evaluated against market-only, market-plus-text, market-plus-sensor, and direct feature concatenation baselines to determine whether infrastructure sensing provides incremental predictive information beyond contemporaneous market activity.

The main contributions of this study are summarized as follows.

1. Infrastructure operating signals are introduced into short-term financial risk sensing and organized with textual and market information in a temporally aligned multimodal dataset. Each prediction sample links financial texts, minute-level infrastructure measurements, historical market variables, and a future 30 min label at a common prediction origin.
2. HSF-LLMNet integrates sensor reliability modeling, learnable asynchronous alignment, and quality-aware multimodal fusion within one framework. Unlike methods that apply reliability weights only during sensor preprocessing, the estimated channel quality affects both hardware temporal encoding and the final fusion gate. Unlike fixed resampling or exact timestamp matching, the asynchronous module learns delay-dependent associations between irregular textual events and continuous sensor sequences. Bidirectional cross-attention then models the asymmetric relationships from semantic events to infrastructure responses and from infrastructure states to semantic context.
3. The incremental predictive value of infrastructure sensing is evaluated against market-only, market-plus-text, market-plus-sensor, and direct feature concatenation baselines. Ablation experiments isolate the effects of sensor reliability estimation, asynchronous alignment, bidirectional interaction, gated fusion, and multitask learning. The study also separates offline LLM extraction from fusion-network inference and reports the parameter count, computational cost, latency, memory use, and edge-deployment performance of the trainable network.

## 2. Related Work

### 2.1. Large Language Models and Sentiment Analysis

Financial sentiment analysis converts news, corporate disclosures, research reports, and social-media content into representations of market expectations, investor sentiment, event type, and policy impact [18,19]. Lexicon-based rules and traditional machine-learning methods are interpretable but have limited capacity to represent context-dependent events [20]. CNNs, recurrent neural networks, and Transformers improve contextual representation and have been used for sentiment classification, event detection, and financial-risk assessment [21,22]. LLMs further support event category extraction, risk intensity estimation, and structured interpretation of financial narratives [14,23,24].

The closest LLM-based financial forecasting studies mainly combine textual representations with structured market variables. For example, the framework in [19] combines financial-news sentiment with structured market data for market sentiment prediction, whereas other studies examine the relationship between LLM-derived semantics and subsequent market behavior [25,26]. These methods do not incorporate trading infrastructure measurements, explicitly estimate sensor channel quality, or learn delays between textual events and infrastructure responses. Text-only or text–market models also remain sensitive to publication delay, duplicated content, and semantic ambiguity. HSF-LLMNet therefore uses calibrated semantic uncertainty to control the contribution of uncertain texts instead of treating all extracted semantic representations as equally reliable.

### 2.2. Hardware Sensors and Intelligent Operational State Perception

Hardware sensing methods use workload, resource utilization, electrical, thermal, network, and vibration measurements to monitor computing systems and edge infrastructures [27,28]. Existing studies primarily target equipment health monitoring, fault diagnosis, anomaly detection, and energy management [29,30]. These studies provide methods for processing missing observations, drift, transient noise, and heterogeneous sensor channels, but their prediction targets are hardware failures or operating states rather than future financial-market outcomes. They are therefore methodological references for sensor representation rather than direct financial forecasting competitors.

In trading infrastructures, server workload, power consumption, and network traffic may change during concentrated market-data requests or order processing [31,32,33]. However, these changes may also be contemporaneous proxies for trading volume rather than leading indicators of future risk. Existing financial forecasting methods rarely evaluate this distinction because they generally exclude infrastructure measurements [34]. HSF-LLMNet models sensor missingness, local drift, abnormality, and channel reliability explicitly [35,36], and evaluates whether sensor inputs provide incremental predictive information after observable market and textual variables have been included.

### 2.3. Multimodal Temporal Fusion and Market Prediction

Multimodal financial forecasting combines text, market time series, macroeconomic variables, and other heterogeneous inputs [37,38]. Existing architectures commonly use feature concatenation, attention, cross-modal Transformers, graph-based interaction, or multi-task learning [39,40]. RiskLabs [40], one of the closest multi-source financial risk frameworks, combines earnings-call information, contextual news, and market time series for multi-task volatility and variance prediction. However, it does not use infrastructure operating signals, model sensor reliability, or estimate event-to-infrastructure response delays. Other text–market fusion studies similarly align inputs through fixed periods, timestamp ordering, or representation-level fusion rather than learnable alignment between irregular events and continuous operational sequences.

The remaining technical gap is therefore not multimodal fusion in general, but the joint treatment of cross-frequency timing, modality quality, and operational sensing. Heterogeneous observations differ in sampling frequency, temporal delay, noise structure, and semantic meaning [41,42]. Existing alignment and fusion methods address some of these differences [43,44,45], but they do not jointly combine infrastructure signals, sensor reliability, calibrated semantic uncertainty, and multi-task short-horizon prediction. HSF-LLMNet uses learnable delay-dependent alignment between irregular texts and one-minute sensor sequences, propagates sensor-reliability estimates into both temporal encoding and gated fusion, and jointly predicts 30 min direction, realized volatility, and risk state. Table 1 provides a structured comparison.

### 2.4. Comparison with Closely Related Studies

As shown in Table 1, existing multimodal financial forecasting methods primarily combine textual information with observable market variables. Some approaches use attention, event-centered aggregation, or representation-level alignment, but they do not jointly incorporate infrastructure operating signals, learnable cross-frequency delay estimation, sensor reliability, and calibrated semantic uncertainty. HSF-LLMNet combines these mechanisms within a shared framework for 30 min direction, realized-volatility, and risk-state prediction.

## 3. Materials and Method

### 3.1. Data Collection

The dataset consists of semantic data, market trading data, and hardware sensor data, as summarized in Table 2. Given that these modalities have different statistical units and temporal coverage, we explicitly distinguish them based on source archive availability, the common multimodal observation interval, and the period used for model development and evaluation. A semantic record denotes one document or investor post, a market record denotes one index–timestamp observation, and a hardware record denotes one timestamped sensor observation. The source archives available for the text and market modalities span January 2022 to December 2025, whereas hardware telemetry is available from 1 March 2024 to 30 June 2025. The December 2025 endpoint therefore refers exclusively to the availability of the complete text and market source archives and does not represent the experimental period used in this study. The common multimodal observation interval was defined at the dataset level as the period during which the text, market, and hardware data sources were jointly available, namely 1 March 2024 to 30 June 2025. Accordingly, all prediction window construction, preprocessing for model inputs, model training, validation, hyperparameter selection, and testing were performed exclusively using prediction origins within this common interval. This dataset-level overlap requirement does not imply that every individual prediction window had to contain a newly published text record; when no index-matched text was available within the textual look-back interval, a learned null-text token was used as described below. Text and market observations outside the common interval were retained only in the source archives for record management and were excluded from prediction sample construction; consequently, they did not contribute to any model input, training or validation procedure, test evaluation, or result reported in this study. The raw and cleaned record counts in Table 2 therefore describe the complete source archives of the corresponding modalities, whereas all aligned-window counts refer exclusively to the common multimodal interval from 1 March 2024 to 30 June 2025.

Semantic data were obtained from Sina Finance, Eastmoney, CNINFO, the information-disclosure platform of the China Securities Regulatory Commission, and five public investor communities. Exact duplicates and cross-platform reproductions were removed according to the associated entity, publication-time proximity, and semantic similarity. When the same announcement or news item appeared on multiple platforms, one canonical record was retained, with the official disclosure source given priority. Distinct investor comments concerning the same event were retained as separate records. The raw collections contained 128,436 news or announcement records and 93,517 investor posts; after abnormal-content removal, timestamp validation, deduplication, and entity matching, 119,842 and 84,963 records remained, respectively. Thus, the cleaned semantic archive contained 204,805 canonical text records in total.

To avoid conflating semantic-document counts with prediction-sample counts, five statistical units are distinguished throughout this study. A raw text record denotes an originally collected document or investor post before cleaning and deduplication. A canonical text record denotes one retained document or post after duplicate removal and source consolidation; the 119,842 news or announcement records and 84,963 investor posts reported above are counts of such canonical records. An index–text association denotes a mapping between one canonical text record and one study index according to the issuer’s constituent membership on the publication date. As one issuer may simultaneously belong to more than one study index, a single canonical record may generate multiple index–text associations without becoming multiple source documents. An event–window membership denotes the inclusion of one index-associated canonical event in the 24-h textual look-back interval of a particular prediction origin. Since the look-back windows roll through time, one index–text association may contribute to multiple consecutive prediction windows. Finally, an index–time prediction window denotes one model sample indexed by $\left(a_{q},T_{q}\right)$ and is the statistical unit used to obtain the final analytical sample size $N_{q}=169,208$. Therefore, the 204,805 canonical semantic records, the number of index–text associations, the number of event–window memberships, and the 169,208 final prediction windows are different quantities and should not be interpreted interchangeably. For example, if an issuer belongs to both the CSI 300 and SSE 50 indices on the publication date, one canonical announcement remains one semantic document but produces two index–text associations. The same announcement may subsequently enter several rolling 24 h textual windows for each associated index, while each $\left(a_{q},T_{q}\right)$ pair still constitutes only one index–time prediction sample. Duplicate event identifiers prevent the same canonical event from being counted more than once within a given index–time window.

The prediction targets were defined at the index level. The market universe consisted of the SSE Composite Index (000001.SH), SSE 50 Index (000016.SH), CSI 300 Index (000300.SH), STAR 50 Index (000688.SH), CSI 500 Index (000905.SH), CSI 1000 Index (000852.SH), Shenzhen Component Index (399001.SZ), and ChiNext Index (399006.SZ). These eight indices were fixed before model development to represent broad-market, large-cap, mid-cap, small-cap, technology-oriented, and growth-oriented segments of the Chinese A-share market. Each prediction sample was indexed by an index $a_{q}$ and a prediction origin $T_{q}$; no stock-level or sector-level forecasts were produced. Company-specific texts were associated with an index according to the issuer’s constituent membership on the publication date. When an issuer belonged to more than one study index, the text could be associated with each relevant index, but duplicate event identifiers prevented repeated use within the same index–time window. References to “representative companies” and unspecified sector-level prediction elsewhere in the manuscript were removed. Market records were obtained from the Shanghai Stock Exchange, Shenzhen Stock Exchange, Yahoo Finance, and Wind Terminal. The complete archive contained 2,517,840 one-minute index–timestamp records, including prices, returns, trading volumes, trading values, realized-volatility variables, and liquidity-related measurements, of which 2,431,776 remained after timestamp reconciliation, missing-record screening, and corporate action or trading calendar checks.

Hardware measurements were collected from a controlled trading-platform simulation using three Dell PowerEdge R750 computing nodes, one NVIDIA Jetson AGX Xavier edge node, INA219 and INA226 electrical monitoring modules, Sensirion SHT35 temperature and relative-humidity sensors, Cisco Catalyst C9300-24T switches, network probes, and MPU6050 inertial sensors. Historical market records were replayed chronologically using their original trading dates and intraday timestamps. At minute *t*, the simulator generated market data queries, mock order submission messages, cancellation requests, and risk-control or account status requests using only information available at or before the end of minute *t*. The request-arrival intensity was specified as

(1) $$\lambda_{t}=\mathrm{clip}\left[\lambda_{0}\left(1+\alpha_{V}z_{V,t}^{+}+\alpha_{R}z_{R,t}^{+}+\alpha_{N}z_{N,t}^{+}\right),\lambda_{\min},\lambda_{\max}\right],$$

where $\lambda_{0}=800$ requests/s, $\lambda_{\min}=200$ requests/s, and $\lambda_{\max}=5000$ requests/s. Let $z_{V,t}$, $z_{R,t}$, and $z_{N,t}$ denote the causally standardized deviations of contemporaneous trading volume, absolute one-minute return, and newly published index-related text count, respectively. Their positive-part transformations are defined as $z_{k,t}^{+}=\max\left(z_{k,t},0\right)$ for k∈{V,R,N}. The standardization parameters were calculated using only the preceding 20 trading days at the same intraday minute. The coefficients were set to $\alpha_{V}=0.55$, $\alpha_{R}=0.30$, and $\alpha_{N}=0.15$. This specification intentionally implements a stress-oriented asymmetric workload generator: Above-reference market activity increases simulated request pressure, whereas below-reference deviations do not reduce the traffic intensity below the baseline solely through these three market-conditioned terms. Consequently, before application of the upper clipping bound, the principal specification satisfies $\lambda_{t}\geq \lambda_{0}=800$ requests/s, and the lower bound $\lambda_{\min}=200$ requests/s is not normally active under this positive-part formulation. It is retained as a numerical safety bound in the simulator rather than as an empirically binding component of the principal workload specification. This distinction is now stated explicitly to avoid interpreting $\lambda_{\min}$ as evidence that the positive-part market-conditioning terms can themselves generate below-baseline traffic.

Generated traffic consisted of 65% market-data queries, 20% order-submission messages, 10% cancellation messages, and 5% risk-control or account-status requests. Thus, simulated workload intensity was partly determined by contemporaneously observable market activity, but it was not generated from the future 30-min return, future realized volatility, future risk state, or any other target variable. This distinction was considered in the incremental-value experiments by controlling for contemporaneous market variables, residualizing sensor features against observable market activity, and excluding the final 5 or 10 min of sensor inputs in the robustness analysis.

For temporal alignment, $T_{q}$ was defined as the end of a completed one-minute interval. Textual records published during the preceding 24 h and market observations from the preceding 60 min were assigned to the corresponding prediction window as

(2) $$D_{q}=\left\{d_{i}|a_{i}∼a_{q},\;T_{q}-24\;h\leq \tau_{i}\leq T_{q}\right\},\;M_{q}=\left\{m_{t}|T_{q}-60\;\min<t\leq T_{q}\right\},$$

where $a_{i}∼a_{q}$ indicates that text $d_{i}$ was associated with index $a_{q}$ and $\tau_{i}$ denotes its publication timestamp. If no associated text was available in $D_{q}$, a learned null-text token was used. Hardware observations from the preceding 60 min and the future observations used to construct the 30 min target were defined as

(3) $$X_{q}^{h}=\left\{x_{t}|T_{q}-60\;\min<t\leq T_{q}\right\},\;y_{q}^{\left(30\right)}=g\left(\left\{P_{t},V_{t}|T_{q}<t\leq T_{q}+30\;\min\right\}\right),$$

where g(·) denotes the target-construction function. Native sensor observations were aggregated into right-aligned one-minute intervals (t−1min,t]. Centered aggregation, backward filling from future observations, and interpolation using values after $T_{q}$ were prohibited. A hardware sequence $X_{q}^{h}$ was considered valid when the corresponding prediction window contained sufficient usable hardware observations, after channel screening, missing-value processing, and one-minute aggregation, to construct the hardware input representation. Simultaneous completeness of every individual hardware modality was not required. Instead, the valid hardware window set was defined as the union of windows for which the retained hardware measurements collectively provided a valid hardware representation.

(4) $$\begin{array}{cc} Q_{30}=\{q=\left(a_{q},T_{q}\right)\;|\; & T_{\mathrm{start}}\leq T_{q}\leq T_{\mathrm{end}},\;y_{q}^{\left(30\right)}\;\mathrm{is}\;\mathrm{available},\\ \; & M_{q}\;\mathrm{is}\;\mathrm{valid},\;X_{q}^{h}\;\mathrm{is}\;\mathrm{valid}\},\\ \; & N_{q}=\left|Q_{30}\right|=169,208. \end{array}$$

where $T_{\mathrm{start}}$ and $T_{\mathrm{end}}$ denote 1 March 2024 and 30 June 2025, respectively. These boundaries correspond to the common multimodal observation interval and therefore define the temporal domain from which all analytical prediction samples were constructed. Under the hardware validity criterion described above, all 169,208 retained prediction windows contained a valid hardware input representation, although the availability count of any individual hardware modality could be smaller than 169,208.

A timestamp-level feature availability audit was conducted after preprocessing and before chronological partitioning. Each text record retained its publication timestamp, and each sensor record retained its replay timestamp, physical acquisition timestamp, and one-minute aggregation endpoint. For every retained prediction window, the following condition was checked:

(5) $$\max\left\{\max_{d_{i}\in D_{q}}\tau_{i},\;\max_{m_{t}\in M_{q}}t,\;\max_{x_{t}\in X_{q}^{h}}t^{\mathrm{replay}},\;\max_{x_{t}\in X_{q}^{h}}t^{\mathrm{acq}},\;\max_{x_{t}\in X_{q}^{h}}t^{\mathrm{agg}}\right\}\leq T_{q}<\min_{u\in Y_{q}}t_{u},$$

where $t^{\mathrm{replay}}$, $t^{\mathrm{acq}}$, and $t^{\mathrm{agg}}$ denote the replay, acquisition, and aggregation end timestamps, and $Y_{q}$ denotes the future observations used to construct the target. A sample was automatically rejected if any input timestamp exceeded $T_{q}$. The target construction procedure was executed only after the input feature cache had been frozen. The audit identified zero feature availability violations among the 169,208 retained windows. The final aligned dataset therefore contained 169,208 index–time prediction windows, consisting of 118,446 training windows, 25,381 validation windows, and 25,381 test windows. The union of windows containing sufficient retained hardware measurements to construct a valid hardware representation corresponded to the same 169,208 prediction windows. As shown in Table 2, semantic aligned window counts indicate prediction windows containing at least one matched canonical record of the corresponding semantic source type, whereas hardware aligned window counts indicate modality-specific hardware availability. These modality-specific counts overlap and therefore should not be summed to obtain $N_{q}$.

### 3.2. Data Augmentation

Data preprocessing and augmentation were performed in a fixed order to prevent duplicate event amplification and information leakage. Event-level deduplication, source consolidation, timestamp standardization, asset matching, and abnormal-record removal were first applied to the complete raw dataset summarized in Table 2. The cleaned records were then divided chronologically into the training, validation, and test portions of each time-series fold. Data augmentation was applied only to the training portion, while preprocessing parameters, augmentation thresholds, and label construction thresholds were estimated exclusively from the corresponding training data.

For the textual modality, let the *i*-th text, its publication time, and associated asset be denoted by $d_{i}$, $\tau_{i}$, and $a_{i}$, respectively. After HTML removal and text normalization, the similarity between two texts was calculated as

(6) $$\mathrm{Sim}\left(d_{i},d_{j}\right)=\frac{e_{i}^{⊤}e_{j}}{∥e_{i}{∥}_{2}{∥e_{j}∥}_{2}},$$

where $e_{i}$ and $e_{j}$ are their semantic embeddings. Two institutional documents were assigned to the same event cluster when they referred to the same asset or industry, their publication times differed by no more than 24 h, and $\mathrm{Sim}(d_{i},d_{j})>0.92$. The same criterion was used to identify reposted investor discussions, whereas distinct comments expressing different opinions about the same event were retained. One canonical record and one event identifier $g_{i}$ were retained for each duplicate cluster. Deduplication and event ID assignment were completed before chronological splitting, ensuring that records associated with the same event could not occur in different data partitions.

The frozen large language model $F_{\mathrm{LLM}}$ produced sentiment polarity, event category, risk intensity, and semantic uncertainty:

(7) $$s_{i}=F_{\mathrm{LLM}}\left(d_{i}\right).$$

Sentiment polarity used the discrete categories positive, neutral, negative, and panic related, whereas semantic uncertainty was an independent scalar $u_{i}\in \left[0,1\right]$. Each canonical text was evaluated through K=3 offline inference rounds, and majority voting was used only for discrete labels:

(8) $${\hat{l}}_{i}=\arg\max_{c}\sum_{k=1}^{K}I\left(l_{i}^{k}=c\right).$$

Texts with calibrated uncertainty $u_{i}>0.70$ were assigned reduced training weights and were excluded from automatic augmentation.

For the hardware modalities, native rate observations were screened using channel-specific physical limits and

(9) $$|x_{t,m}-\mu_{m}|>k\sigma_{m},\;k=3,$$

where $\mu_{m}$ and $\sigma_{m}$ were estimated from the training portion of the current fold. The valid native observations were divided into non-overlapping one-minute intervals $\Omega_{r}$. For channel *m*, the minute-level level and fluctuation descriptors were defined as

(10) $$h_{r,m}=\left[{\bar{x}}_{r,m},s_{r,m},x_{r,m}^{\min},x_{r,m}^{\max},\kappa_{r,m},\eta_{r,m}\right],$$

(11) $$\begin{array}{cc} {\bar{x}}_{r,m}\; & =\frac{1}{n_{r,m}}\sum_{t\in \Omega_{r}}x_{t,m},\\ s_{r,m}\; & =\sqrt{\frac{1}{n_{r,m}}\sum_{t\in \Omega_{r}}{\left(x_{t,m}-{\bar{x}}_{r,m}\right)}^{2}},\\ \eta_{r,m}\; & =\frac{1}{n_{r,m}-1}\sum_{j=2}^{n_{r,m}}\left|x_{j,m}-x_{j-1,m}\right|. \end{array}$$

where $\kappa_{r,m}$ is the least-squares intraminute slope. A minute was considered valid when at least 80% of its expected native observations remained after anomaly filtering.

Missing minute-level vectors were numerically completed while their missingness patterns were retained. Let $M_{r,m}=1$ indicate a valid minute and $M_{r,m}=0$ indicate a missing or insufficiently sampled minute. Gaps of at most $G_{\max}=5$ min were linearly interpolated. For longer gaps, the latest valid vector was carried forward for no more than $F_{\max}=2$ min, after which the training set median vector ${\tilde{\mu }}_{m}$ was used:

(12) $$h_{r,m}^{\mathrm{num}}=\left\{\begin{array}{cc} h_{r,m},\; & M_{r,m}=1,\\ h_{a,m}+\frac{r-a}{b-a}\left(h_{b,m}-h_{a,m}\right),\; & M_{r,m}=0,\;b-a\leq G_{\max},\\ h_{a,m},\; & M_{r,m}=0,\;b-a>G_{\max},\;r-a\leq F_{\max},\\ {\tilde{\mu }}_{m},\; & \mathrm{otherwise}, \end{array}\right.$$

where *a* and *b* are the nearest valid minute indices before and after *r*. Numerically completed positions remained marked by $M_{r,m}=0$, and $\Delta_{r,m}$ recorded the elapsed time since the latest valid observation.

The previous moving mean subtraction was replaced by causal local detrending. It was not assumed that the estimated baseline represented only device drift; instead, the original signal level, estimated baseline, and residual were all retained. For channel *m*, the baseline was estimated from originally valid observations in the preceding $L_{b}=240$ min using a robust causal median:

(13) $$b_{r,m}=\mathrm{median}\left\{{\bar{x}}_{j,m}|r-L_{b}+1\leq j\leq r,\;M_{j,m}=1\right\},\;L_{b}=240.$$

If fewer than $N_{b}=30$ valid observations were available, the latest estimated baseline was carried forward. The detrended residual was defined as

(14) $$e_{r,m}={\bar{x}}_{r,m}^{\mathrm{num}}-b_{r,m}.$$

The changes in the original level, baseline, and residual were calculated separately:

(15) $$\Delta {\bar{x}}_{r,m}={\bar{x}}_{r,m}^{\mathrm{num}}-{\bar{x}}_{r-1,m}^{\mathrm{num}},\;\Delta b_{r,m}=b_{r,m}-b_{r-1,m},\;\Delta e_{r,m}=e_{r,m}-e_{r-1,m}.$$

The final representation of channel *m* at minute *r* was

(16) $$f_{r,m}=\left[h_{r,m}^{\mathrm{num}}∥b_{r,m}∥e_{r,m}∥\Delta {\bar{x}}_{r,m}∥\Delta b_{r,m}∥\Delta e_{r,m}∥M_{r,m}∥\log\left(1+\Delta_{r,m}\right)\right].$$

All feature dimensions were standardized using training set statistics. Consequently, local detrending did not replace or destroy the slowly varying signal level. For temperature, humidity, and other channels whose gradual variation may represent a physical response, the model could use the original level and baseline trend in addition to the residual. The resulting level–baseline–residual representation was supplied to the hardware temporal module shown in Figure 2.

For the principal 30 min task, each sample was indexed by asset $a_{q}$ and prediction origin $T_{q}$. The textual and hardware windows shared the same right endpoint but could have different durations:

(17) $$D_{q}=\left\{d_{i}|a_{i}=a_{q},\;T_{q}-L_{d}\leq \tau_{i}\leq T_{q}\right\},$$

(18) $$X_{q}=\left\{f_{r}|T_{q}-L_{x}\leq T_{r}\leq T_{q}\right\}.$$

The final values were $L_{d}=24$ h and $L_{x}=60$ min, selected using the training and validation portions as reported in Section 4.6. If no asset-matched text was available, a learned null-text token was used. If more than 64 deduplicated events occurred, the 64 most recent events were retained. A hardware window was valid when at least 48 of its 60 min-level vectors were available.

All input information was required to be available no later than $T_{q}$. The model performed three parallel tasks over the same future interval

(19) $$H_{q}=\left(T_{q},T_{q}+\Delta T\right],\;\Delta T=30\;\min,$$

including direction classification, realized volatility regression, and three-level risk classification. These targets were constructed independently from observations within $H_{q}$; the volatility and risk targets were not derived from the binary direction label.

For the direction task, the future simple return and binary label were defined as

(20) $$\begin{array}{cc} r_{q}\; & =\frac{P_{T_{q}+\Delta T}-P_{T_{q}}}{P_{T_{q}}},\\ y_{q}^{\mathrm{dir}}\; & =I\left(r_{q}>\delta \right),\;\delta =5\times 10^{-4}. \end{array}$$

where δ=0.0005 corresponds to 5 basis points. For the volatility task, the future 30 min interval was divided into H=30 one-minute subintervals. Let

(21) $$g_{q,\ell }=\log\left(\frac{P_{T_{q}+\ell \Delta \tau }}{P_{T_{q}+\left(\ell -1\right)\Delta \tau }}\right),\;\Delta \tau =1\;\min,\;\ell =1,\ldots ,H,$$

denote the one-minute log return. The realized volatility regression target was

(22) $$y_{q}^{\mathrm{vol}}=\nu_{q}=\sqrt{\sum_{\ell =1}^{H}g_{q,\ell }^{2}}.$$

The risk level target jointly considered realized volatility, absolute return, and liquidity stress. The absolute return indicator was $\xi_{q}=\left|r_{q}\right|$. Let $V_{q,\ell }$ denote the monetary trading volume in future minute *ℓ*, normalized by the median one-minute volume of asset $a_{q}$ in the corresponding training portion:

(23) $${\tilde{V}}_{q,\ell }=\frac{V_{q,\ell }}{\mathrm{median}\left(V_{\mathrm{train},a_{q}}\right)+\varepsilon },\;\varepsilon =10^{-8}.$$

The liquidity stress indicator was defined as

(24) $$\ell_{q}=\frac{1}{H}\sum_{\ell =1}^{H}\frac{|g_{q,\ell }|}{{\tilde{V}}_{q,\ell }+\varepsilon }.$$

For each fold *f*, the 60th, 85th, and 95th percentile thresholds of $\nu_{q}$, $\xi_{q}$, and $\ell_{q}$ were estimated exclusively from its training portion:

(25) $$\theta_{z,p}^{\left(f\right)}={\mathrm{Quantile}}_{p}\left(\left\{z_{q}|q\in Q_{\mathrm{train}}^{\left(f\right)}\right\}\right),\;z\in \left\{\nu ,\xi ,\ell \right\},\;p\in \left\{0.60,0.85,0.95\right\}.$$

Define

(26) $$I_{q,z}^{\left(p\right)}=I\left(z_{q}\geq \theta_{z,p}^{\left(f\right)}\right).$$

The risk label was then constructed as

(27) $$y_{q}^{\mathrm{risk}}=\left\{\begin{array}{cc} 2,\; & \sum_{z\in \{\nu ,\xi ,\ell \}}I_{q,z}^{\left(0.85\right)}\geq 2\;\mathrm{or}\;\sum_{z\in \{\nu ,\xi ,\ell \}}I_{q,z}^{\left(0.95\right)}\geq 1,\\ 1,\; & \sum_{z\in \{\nu ,\xi ,\ell \}}I_{q,z}^{\left(0.60\right)}\geq 2\;\mathrm{or}\;\sum_{z\in \{\nu ,\xi ,\ell \}}I_{q,z}^{\left(0.85\right)}\geq 1,\\ 0,\; & \mathrm{otherwise}, \end{array}\right.$$

where labels 0, 1, and 2 represent low, medium, and high risk, respectively. The high-risk condition was evaluated first, and the medium-risk rule was applied only to samples not assigned to the high-risk class.

In the aligned dataset, the direction task contained 86,431 non-upward or approximately flat samples and 82,777 upward samples. The low-, medium-, and high-risk classes contained 100,884, 42,735, and 25,589 samples, respectively. Risk class imbalance was handled using fold-specific weighted cross-entropy:

(28) $$w_{c}^{\left(f\right)}=\frac{N_{\mathrm{train}}^{\left(f\right)}}{3N_{c,\mathrm{train}}^{\left(f\right)}},\;c\in \left\{0,1,2\right\},$$

where the weights were calculated only from the training portion. The dependence analysis confirmed that the tasks were related but not nested. Across the five folds, the point-biserial correlation between the direction label and realized volatility was 0.08±0.02, Cramér’s *V* between direction and risk level was 0.11±0.02, and the Spearman correlation between realized volatility and risk level was 0.76±0.03. The stronger association between volatility and risk occurred because realized volatility was one component of the risk definition; however, the risk label additionally incorporated absolute return and liquidity stress. The three prediction heads therefore estimated $y_{q}^{\mathrm{dir}}$, $y_{q}^{\mathrm{vol}}$, and $y_{q}^{\mathrm{risk}}$ as parallel outputs rather than sequential or nested predictions.

Text augmentation was performed only within the training portion of each fold. Its purpose was to generate alternative surface expressions of an existing canonical event rather than to synthesize new market events. Minority sentiment classes were augmented by increasing the sampling probability of their existing canonical texts from 0.15 to 0.30; no synthetic asset, organization, numerical value, or event outcome was introduced solely to balance the class distribution.

Two controlled augmentation operators were used. Synonym-based paraphrasing replaced only non-entity, non-numeric, and non-sentiment-bearing tokens using a financial domain synonym lexicon. Asset names, organization names, dates, numerical values, measurement units, negation terms, modal expressions, causal terms, and sentiment-bearing keywords were protected from replacement. No more than 10% of the replaceable tokens, with an upper limit of five replacements per text, were modified. Event template rewriting used a fixed library of 12 grammatical templates to change the surface form while preserving the asset, organization, event type, action, numerical value, unit, publication time, impact direction, and sentiment polarity. The templates were not permitted to introduce a new entity, numerical claim, causal relationship, or event outcome.

For the *j*-th candidate generated from canonical text $d_{i}$, the augmentation operation was denoted by

(29) $$d_{i,j}^{\mathrm{aug}}=T_{\mathrm{text}}^{\left(j\right)}\left(d_{i}\right).$$

A candidate was retained only when all protected factual slots exactly matched those of the canonical text and its semantic similarity satisfied

(30) $$0.85\leq \mathrm{Sim}\left(d_{i},d_{i,j}^{\mathrm{aug}}\right)\leq 0.98.$$

**Figure 1:**
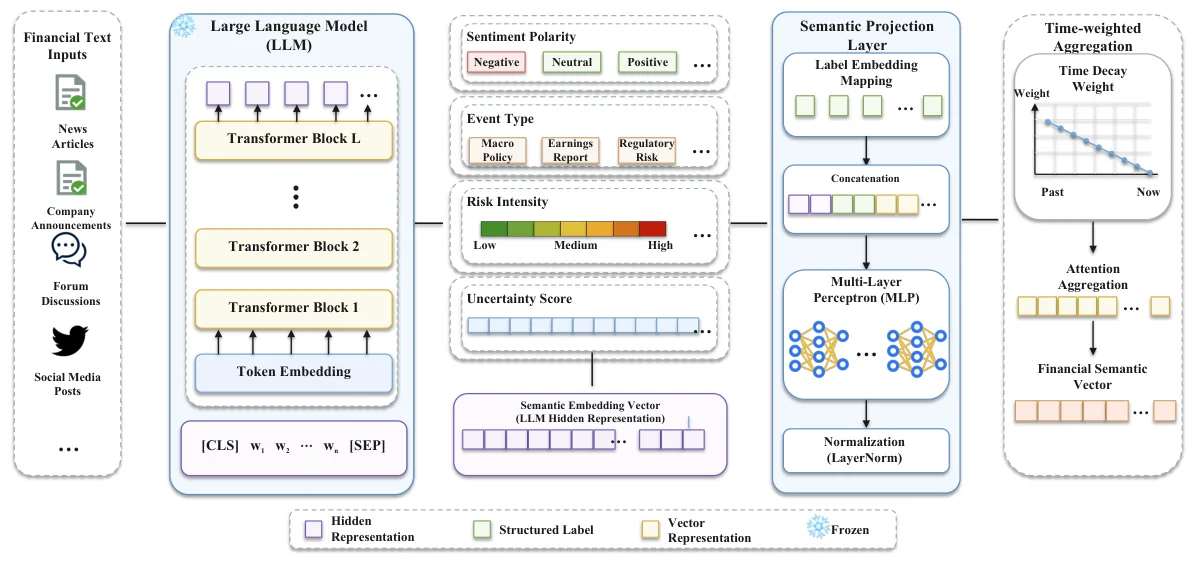
Architecture of the LLM-driven semantic sentiment encoding module. The frozen LLM produces a contextual representation, while separate prediction heads estimate sentiment polarity, event category, risk intensity, and semantic uncertainty. Risk intensity increases the aggregation relevance of high-impact texts, whereas semantic uncertainty reduces the contribution of ambiguously interpreted texts.

The lower bound rejected semantic drift, whereas the upper bound removed nearly identical copies. Bidirectional textual entailment was additionally required:

(31) $$\min\left\{p_{\mathrm{ent}}\left(d_{i}\Rightarrow d_{i,j}^{\mathrm{aug}}\right),p_{\mathrm{ent}}\left(d_{i,j}^{\mathrm{aug}}\Rightarrow d_{i}\right)\right\}\geq 0.85,$$

where $p_{\mathrm{ent}}\left(\cdot \right)$ denotes the entailment probability produced by the frozen semantic validation model. The sentiment polarity, event category, and impact direction of every candidate were re-evaluated using the semantic encoding procedure shown in Figure 1. A candidate was accepted only when all three discrete outputs were identical to those of the canonical text. Candidates with calibrated semantic uncertainty greater than $\rho_{u}=0.70$ were rejected. At most two validated variants were retained for each canonical text:

(32) $$\begin{array}{cc} 0\; & \leq A_{i}\leq A_{\max},\\ A_{\max}\; & =2. \end{array}$$

The semantic equivalence, sentiment preservation, factual consistency, and scenario realism assessments of the accepted augmented texts are reported in Section 4.8.

All accepted variants inherited the event identifier, asset identifier, publication time, and prediction targets of their canonical text. To prevent augmentation from increasing the total contribution of an event, the original text and its accepted variants shared one normalized event-level weight:

(33) $${\bar{s}}_{g}=\sum_{j=0}^{A_{g}}\beta_{g,j}F_{\mathrm{LLM}}\left(d_{g}^{\left(j\right)}\right),\;\beta_{g,j}=\frac{1}{A_{g}+1},\;\sum_{j=0}^{A_{g}}\beta_{g,j}=1.$$

The window-level textual representation was aggregated over unique event identifiers:

(34) $$Z_{q}^{\mathrm{text}}=\sum_{g\in G_{q}}\omega_{q,g}{\bar{s}}_{g},\;\sum_{g\in G_{q}}\omega_{q,g}=1,$$

where $G_{q}$ denotes the set of unique events within prediction window *q*. Consequently, augmentation could improve robustness to linguistic variation but could not create additional event-level evidence. Validation and test sets contained only cleaned canonical texts. Hardware augmentation was generated online only from the minute-level training sequences. Random masking removed 5% of time–feature positions, sensor channel dropout used p=0.10, Gaussian noise used $\sigma_{\epsilon ,m}=0.05\sigma_{m}^{\mathrm{train}}$, and temporal perturbation was restricted to ±2 one-minute intervals:

(35) $$\tilde{X}=X⊙Q,\;Q\in {\left\{0,1\right\}}^{T\times M},$$

(36) $${\tilde{x}}_{r,m}=x_{r,m}+\epsilon_{r,m},\;\epsilon_{r,m}∼N\left(0,\sigma_{\epsilon ,m}^{2}\right),\;\sigma_{\epsilon ,m}=0.05\sigma_{m}^{\mathrm{train}},$$

(37) $${\tilde{x}}_{r,m}=q_{m}x_{r,m},\;q_{m}∼\mathrm{Bernoulli}\left(1-p\right),\;p=0.10,$$

(38) $${\tilde{x}}_{r,m}=x_{\phi (r),m},\;\left|\phi \left(r\right)-r\right|\leq 2,$$

where ϕ(r) is a monotonic mapping over the one-minute timeline. No augmentation was applied to validation or test sequences, and temporal perturbation was not permitted to move observations beyond the prediction origin $T_{q}$.

### 3.3. Proposed Method

#### 3.3.1. Overall

A hardware sensor and large language model semantic fusion network, termed HSF-LLMNet, is proposed to jointly model semantic events and the operational states of trading infrastructures for short-term market direction prediction, volatility estimation, and risk-level classification. Time-matched text sequences and multichannel hardware sensor sequences are provided as model inputs, and forward propagation is conducted through five successive stages: unimodal encoding, temporal alignment, cross-modal interaction, reliability-aware fusion, and multi-task prediction. First, texts are processed by the LLM-driven semantic sentiment encoding module. Contextual semantic representations are extracted from each text, while structured attributes, including sentiment polarity, event category, impact direction, risk intensity, and semantic uncertainty, are simultaneously generated. Different semantic attributes are embedded and projected into a unified feature space. Time decay weighting and attention-based aggregation are then performed according to publication time, event impact, and semantic confidence, producing a window-level semantic representation.

Meanwhile, multichannel hardware sensor sequences are processed by the reliable hardware sensor temporal modeling module. Dynamic reliability scores are first estimated according to the missing ratio, local stability, abnormal fluctuations, and historical consistency of each sensor channel. These scores are subsequently used to adaptively regulate the contribution of different hardware channels. The reliability-weighted sensor sequences are processed by multiscale temporal convolutions to extract short-term local variations. A temporal Transformer is then employed to model long-range dependencies among power consumption, temperature, device workload, network latency, packet loss rate, and other operational measurements. Consequently, both time-step-level hardware state features and a window-level infrastructure pressure representation are obtained. The two modalities are subsequently introduced into the asynchronous cross-modal fusion module. Since events occur at low and irregular frequencies, whereas hardware signals are continuously collected at high frequencies, semantic representations are first softly aligned with a unified temporal axis according to text publication timestamps and potential response delays in hardware operational states. After temporal alignment, bidirectional cross-attention is employed to facilitate semantic–hardware interaction. Semantic-to-hardware attention is used to identify infrastructure state changes associated with specific events, whereas hardware-to-semantic attention is used to determine which market events can explain the observed system pressure. The resulting cross-modal interaction features and the two unimodal representations are jointly provided to a gated fusion network. The gating coefficients are dynamically determined by semantic uncertainty, sensor reliability, and cross-modal consistency. Consequently, the semantic branch is strengthened when sufficient and reliable textual information is available, whereas the hardware branch receives greater emphasis when textual information is sparse or noisy. Finally, the fused representation is delivered to a direction classification head, a volatility regression head, and a risk-level classification head. Future market states are predicted through multi-task joint learning.

#### 3.3.2. LLM-Driven Semantic Sentiment Encoding Module

The semantic sentiment encoding module takes the sequence of texts within a prediction window as input. It separately represents discrete sentiment polarity, event category, continuous risk intensity, and continuous semantic uncertainty. Sentiment polarity is classified into positive, neutral, negative, and panic-related states, whereas semantic uncertainty is represented by an independent scalar $u_{i}\in \left[0,1\right]$. A value close to zero indicates that the semantic interpretation is stable and unambiguous, while a value close to one indicates substantial disagreement, ambiguity, or insufficient contextual evidence. Therefore, uncertainty is not included in the sentiment category set.

As shown in Figure 1, let $d_{l}$ denote the LLM hidden dimension, $h_{l}$ denote the number of attention heads, $d_{h}=d_{l}/h_{l}$ denote the feature dimension of each head, $d_{f}$ denote the feed-forward dimension, and $N_{l}$ denote the number of Transformer layers. For the *i*-th text, tokenization produces a sequence of length $L_{i}$. Token, positional, and source embeddings are combined as

(39) $$H_{i}^{\left(0\right)}=E_{\mathrm{tok}}\left(x_{i}\right)+E_{\mathrm{pos}}\left(p_{i}\right)+E_{\mathrm{src}}\left(s_{i}\right),\;H_{i}^{\left(0\right)}\in R^{L_{i}\times d_{l}}.$$

In the *n*-th semantic encoding layer, the query, key, and value matrices of attention head *m* are

(40) $$Q_{i,m}^{\left(n\right)}=H_{i}^{\left(n-1\right)}W_{m}^{Q},\;K_{i,m}^{\left(n\right)}=H_{i}^{\left(n-1\right)}W_{m}^{K},\;V_{i,m}^{\left(n\right)}=H_{i}^{\left(n-1\right)}W_{m}^{V}.$$

Here, $W_{m}^{Q},W_{m}^{K},W_{m}^{V}\in R^{d_{l}\times d_{h}}$. The multi-head attention output is

(41) $$A_{i}^{\left(n\right)}={\mathrm{Concat}}_{m=1}^{h_{l}}\left[\mathrm{Softmax}\left(\frac{Q_{i,m}^{\left(n\right)}K_{i,m}^{(n)⊤}}{\sqrt{d_{h}}}\right)V_{i,m}^{\left(n\right)}\right]W^{O}.$$

Residual connections, layer normalization, and a two-layer feed-forward network are then applied:

(42) $${\tilde{H}}_{i}^{\left(n\right)}=\mathrm{LN}\left(H_{i}^{\left(n-1\right)}+A_{i}^{\left(n\right)}\right),$$

(43) $$H_{i}^{\left(n\right)}=\mathrm{LN}\left[{\tilde{H}}_{i}^{\left(n\right)}+\sigma \left({\tilde{H}}_{i}^{\left(n\right)}W_{1}^{\left(n\right)}+b_{1}^{\left(n\right)}\right)W_{2}^{\left(n\right)}+b_{2}^{\left(n\right)}\right].$$

After $N_{l}$ layers, semantic attention pooling is used to obtain the contextual representation:

(44) $$\alpha_{i,j}=\frac{\exp\left[u_{s}^{⊤}\tanh\left(W_{s}h_{i,j}^{\left(N_{l}\right)}+b_{s}\right)\right]}{\sum_{r=1}^{L_{i}}\exp\left[u_{s}^{⊤}\tanh\left(W_{s}h_{i,r}^{\left(N_{l}\right)}+b_{s}\right)\right]},$$

(45) $$z_{i}^{\mathrm{ctx}}=\sum_{j=1}^{L_{i}}\alpha_{i,j}h_{i,j}^{\left(N_{l}\right)}.$$

Four parallel heads are constructed from $z_{i}^{\mathrm{ctx}}$. Sentiment polarity and event category are predicted as discrete probability vectors, whereas risk intensity and semantic uncertainty are estimated as continuous values:

(46) $$p_{i}^{\mathrm{sen}}=\mathrm{Softmax}\left[W_{\mathrm{sen}}\sigma \left(W_{p}z_{i}^{\mathrm{ctx}}+b_{p}\right)+b_{\mathrm{sen}}\right],$$

(47) $$p_{i}^{\mathrm{evt}}=\mathrm{Softmax}\left[W_{\mathrm{evt}}\sigma \left(W_{p}z_{i}^{\mathrm{ctx}}+b_{p}\right)+b_{\mathrm{evt}}\right],$$

(48) $$r_{i}=\mathrm{Sigmoid}\left[w_{r}^{⊤}\sigma \left(W_{r}z_{i}^{\mathrm{ctx}}+b_{r}\right)+b_{r}\right],\;r_{i}\in \left[0,1\right],$$

(49) $$u_{i}^{\mathrm{raw}}=\mathrm{Sigmoid}\left[w_{u}^{⊤}\sigma \left(W_{u}z_{i}^{\mathrm{ctx}}+b_{u}\right)+b_{u}\right],\;u_{i}^{\mathrm{raw}}\in \left[0,1\right].$$

The uncertainty head was trained using a target constructed independently of the window-level aggregation process. Let $p_{i}^{\left(k\right)}$ denote the class probability vector generated in inference round *k*, and let

(50) $${\bar{p}}_{i}=\frac{1}{K}\sum_{k=1}^{K}p_{i}^{\left(k\right)}$$

denote the averaged distribution. The round-level label-disagreement ratio and normalized output entropy were calculated as

(51) $$d_{i}^{\mathrm{dis}}=1-\frac{1}{K}\max_{c}\sum_{k=1}^{K}I\left(l_{i}^{\left(k\right)}=c\right),$$

(52) $$h_{i}^{\mathrm{ent}}=-\frac{1}{\log C}\sum_{c=1}^{C}{\bar{p}}_{i,c}\log\left({\bar{p}}_{i,c}+\varepsilon \right),$$

where *C* is the number of discrete semantic classes and $\varepsilon =10^{-8}$ prevents numerical instability.

A stratified subset of 2000 texts was independently assessed by two annotators with financial domain experience. Each text was assigned one of five semantic uncertainty levels, {0,0.25,0.50,0.75,1.00}, based on ambiguity in event attribution, sentiment direction, causal interpretation, and contextual completeness. Disagreements exceeding one level were resolved by a third annotator. Let $a_{i}^{\mathrm{hum}}$ denote the averaged human uncertainty score. For annotated texts, the uncertainty target was defined as

(53) $$u_{i}^{\mathrm{tar}}=0.35d_{i}^{\mathrm{dis}}+0.35h_{i}^{\mathrm{ent}}+0.30a_{i}^{\mathrm{hum}}.$$

For texts without human annotations, the target was defined as

(54) $$u_{i}^{\mathrm{tar}}=0.50d_{i}^{\mathrm{dis}}+0.50h_{i}^{\mathrm{ent}}.$$

The uncertainty head was optimized using the Huber loss between $u_{i}^{\mathrm{raw}}$ and $u_{i}^{\mathrm{tar}}$. Isotonic regression fitted only on the calibration portion of each training fold was subsequently applied:

(55) $$u_{i}=\mathrm{Cal}\left(u_{i}^{\mathrm{raw}}\right),\;u_{i}\in \left[0,1\right].$$

Table 3 reports the agreement and calibration performance of the uncertainty estimator. The values are reported as mean ± standard deviation across the five time-series folds.

As shown in Table 3, the uncertainty estimator agreed with the human judgments, and isotonic calibration reduced the expected calibration error. The uncertainty value $u_{i}$ is computed from the contextual representation of an individual text before any multi-text aggregation is performed. It therefore does not depend on the window-level representation or aggregation weight.

To separate semantic content from the two scalar aggregation controls, the contextual representation, sentiment probabilities, and event category probabilities are projected into a semantic content space:

(56) $$z_{i}^{\mathrm{sem}}=\mathrm{LN}\left[W_{c}\left(z_{i}^{\mathrm{ctx}}∥p_{i}^{\mathrm{sen}}∥p_{i}^{\mathrm{evt}}\right)+b_{c}\right].$$

Risk intensity $r_{i}$ and calibrated uncertainty $u_{i}$ are not embedded again in the semantic content term. Instead, they enter the aggregation score through separate additive terms, which makes their effects explicit and avoids an unintended multiplicative interaction.

For multiple texts within the same prediction window, the unnormalized aggregation score is defined as

(57) $$e_{i}=-\lambda \Delta t_{i}+\eta_{r}r_{i}-\eta_{u}u_{i}+q^{⊤}\tanh\left(W_{a}z_{i}^{\mathrm{sem}}\right),$$

where λ≥0, $\eta_{r}\geq 0$, and $\eta_{u}\geq 0$. The four terms respectively represent temporal decay, event risk intensity, semantic uncertainty suppression, and learned semantic relevance. The normalized aggregation weight is

(58) $$\omega_{i}=\frac{\exp\left(-\lambda \Delta t_{i}+\eta_{r}r_{i}-\eta_{u}u_{i}+q^{⊤}\tanh\left(W_{a}z_{i}^{\mathrm{sem}}\right)\right)}{\sum_{k=1}^{T_{s}}\exp\left(-\lambda \Delta t_{k}+\eta_{r}r_{k}-\eta_{u}u_{k}+q^{⊤}\tanh\left(W_{a}z_{k}^{\mathrm{sem}}\right)\right)}.$$

The window-level textual representation is then

(59) $$Z_{t}^{\mathrm{LLM}}=\sum_{i=1}^{T_{s}}\omega_{i}z_{i}^{\mathrm{sem}}.$$

Equation (58) replaces the ambiguous product $\eta_{r}r_{i}\eta_{u}u_{i}$ in the original manuscript. Risk intensity and uncertainty are not multiplied. The implemented aggregation layer uses the same additive expression as Equation (57): a larger $r_{i}$ increases the logit by $\eta_{r}r_{i}$, whereas a larger $u_{i}$ decreases it by $\eta_{u}u_{i}$. The manuscript equations and the aggregation code were checked to ensure that this correction was applied to both the mathematical description and the reported experiments.

As $\omega_{i}\geq 0$ and $\sum_{i}\omega_{i}=1$, the aggregated representation lies within the convex hull of the individual semantic vectors. For fixed aggregation weights, a perturbation $\delta_{i}$ satisfies

(60) $${∥\Delta Z_{t}^{\mathrm{LLM}}∥}_{2}={∥\sum_{i=1}^{T_{s}}\omega_{i}\delta_{i}∥}_{2}\leq \sum_{i=1}^{T_{s}}\omega_{i}{∥\delta_{i}∥}_{2}\leq \max_{1\leq i\leq T_{s}}{∥\delta_{i}∥}_{2}.$$

Since $z_{i}^{\mathrm{sem}}$ does not contain $u_{i}$, the effect of uncertainty on the corresponding aggregation weight is

(61) $$\frac{\partial \omega_{i}}{\partial u_{i}}=-\eta_{u}\omega_{i}\left(1-\omega_{i}\right)\leq 0.$$

Similarly, the effect of risk intensity is

(62) $$\frac{\partial \omega_{i}}{\partial r_{i}}=\eta_{r}\omega_{i}\left(1-\omega_{i}\right)\geq 0.$$

Thus, greater calibrated uncertainty cannot increase the aggregation weight when the remaining inputs are fixed, whereas greater estimated risk intensity cannot reduce it. Risk intensity, semantic uncertainty, and temporal decay therefore have distinct roles: $r_{i}$ represents the estimated severity of the event, $u_{i}$ represents the reliability of its semantic interpretation, and $\Delta t_{i}$ represents its temporal relevance. This formulation allows high-risk and clearly expressed events to receive larger weights while suppressing ambiguous, inconsistent, or outdated texts.

#### 3.3.3. Hardware Sensor-Aware Reliable Temporal Modeling Module

The hardware sensor-aware reliable temporal modeling module receives the level–baseline–residual feature sequence defined in Equation (16). The original minute-level signal, causal baseline, detrended residual, and their respective changes are retained as separate inputs. Local detrending is therefore used as feature decomposition rather than as irreversible drift removal.

**Figure 2:**
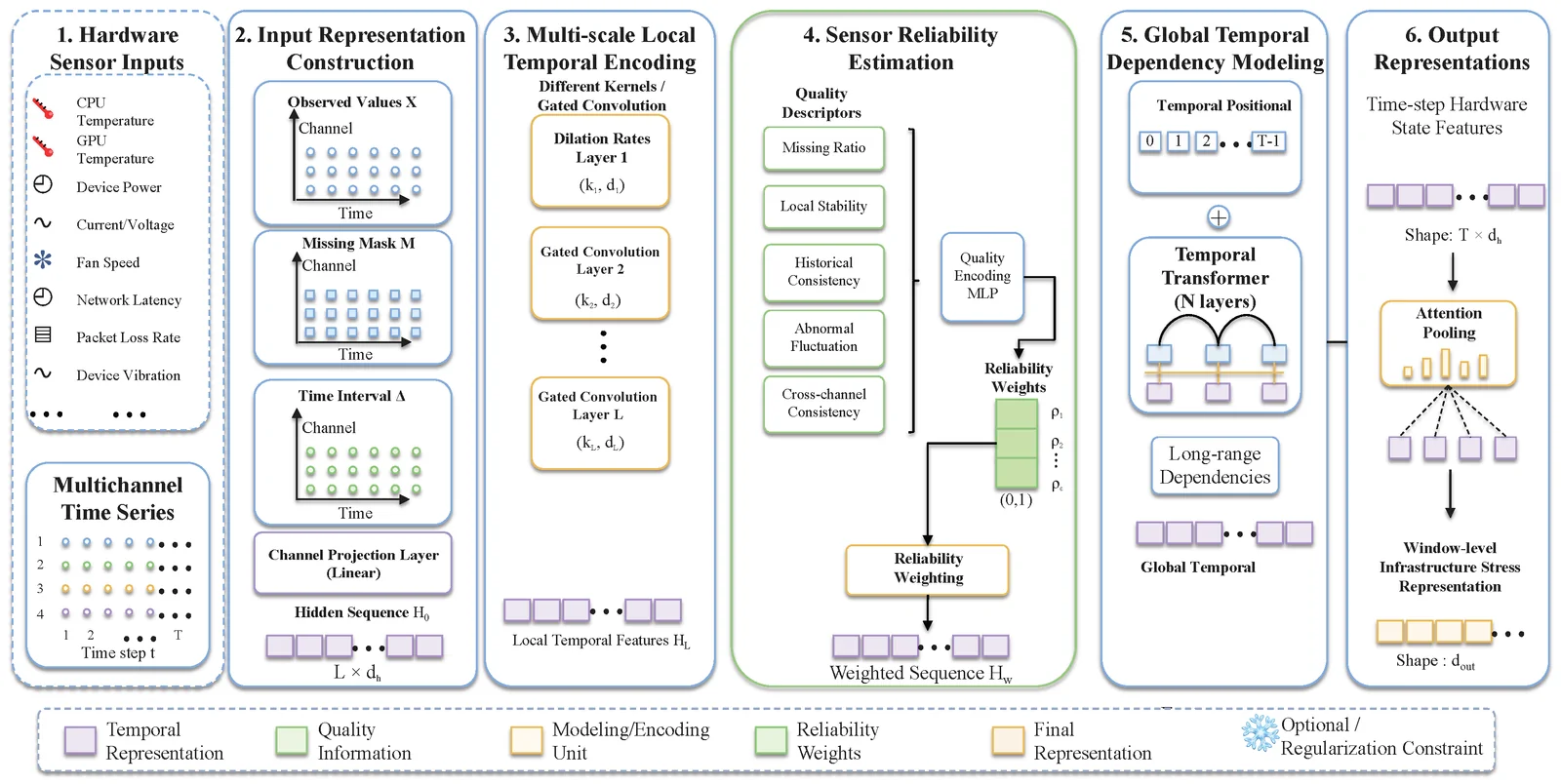
Architecture of the reliable hardware sensor temporal modeling module. The input contains the original sensor level, causal baseline, detrended residual, level and baseline changes, missingness indicators, and elapsed-time variables, followed by multiscale temporal encoding, sensor reliability estimation, and global dependency learning.

As shown in Figure 2, the input sequence is denoted by $X^{h}\in R^{T_{h}\times C_{h}^{*}}$, where $C_{h}^{*}$ includes the expanded level, baseline, residual, change-rate, missing-mask, and elapsed-time features. A projection layer maps $X^{h}$ into the hidden space before multiscale causal convolution and reliability-aware temporal modeling. As the original level and baseline trend remain available, the module can distinguish abrupt residual deviations from gradual thermal, environmental, or workload-related changes rather than treating every slow variation as sensor drift. A missing-value mask $M^{h}\in {\left\{0,1\right\}}^{T_{h}\times C_{h}}$ is retained, where $M_{t,c}^{h}=1$ indicates an originally valid observation and $M_{t,c}^{h}=0$ indicates a missing, invalid, or numerically completed value. The elapsed-time matrix $\Delta^{h}\in R_{\geq 0}^{T_{h}\times C_{h}}$ records the number of minutes since the most recent valid observation. The model input is constructed as

(63) $$U^{h}=X^{h,\mathrm{num}}∥M^{h}∥\log\left(1+\frac{\Delta^{h}}{G_{\max}}\right),\;U^{h}\in R^{T_{h}\times 3C_{h}},$$

where ‖ denotes feature concatenation. Thus, $X^{h,\mathrm{num}}$ provides finite numerical inputs, whereas $M^{h}$ and $\Delta^{h}$ preserve the location and duration of missing observations.

A linear projection maps the input dimension from $3C_{h}$ to $D_{h}$:

(64) $$F^{\left(0\right)}=U^{h}W_{\mathrm{in}}+b_{\mathrm{in}},\;F^{\left(0\right)}\in R^{T_{h}\times D_{h}}.$$

The projected sequence is processed by $N_{c}$ gated causal convolutional layers. For layer *l*, the local response and gate are defined as

(65) $$P^{\left(l\right)}=\tanh\left[{Conv1D}_{K_{l},R_{l}}\left(F^{\left(l-1\right)};W_{p}^{\left(l\right)}\right)\right],$$

(66) $$G^{\left(l\right)}=\mathrm{Sigmoid}\left[{Conv1D}_{K_{l},R_{l}}\left(F^{\left(l-1\right)};W_{g}^{\left(l\right)}\right)\right],$$

(67) $$F^{\left(l\right)}=\mathrm{Norm}\left[F^{\left(l-1\right)}+P^{\left(l\right)}⊙G^{\left(l\right)}\right].$$

The effective receptive field is

(68) $$R_{\mathrm{eff}}=1+\sum_{l=1}^{N_{c}}\left(K_{l}-1\right)R_{l}.$$

This encoder models intraminute summaries of network jitter and power fluctuations together with slower workload and thermal state changes without introducing a separate native-frequency input branch.

After local temporal encoding, the reliability of each channel is estimated once from descriptors computed before reliability weighting. For channel *c*, a fixed related channel set N(c) is specified according to the acquisition variables and physical relationships. The sets include CPU utilization–CPU power, GPU utilization–GPU power, device temperature–fan speed, current–voltage–power, and throughput–port traffic–packet-loss relationships. These sets are fixed before training and do not depend on the estimated reliability scores.

For each related pair (c,j), a robust Spearman correlation ${\hat{\varrho }}_{c,j}$ is calculated using only timestamps at which both channels are originally valid. Let $s_{c,j}\in \left\{-1,1\right\}$ denote the expected correlation sign, where $s_{c,j}=1$ is used for utilization–power, temperature–fan speed, and throughput–port traffic, while $s_{c,j}=-1$ is used for throughput–packet loss. The robust association score is

(69) $$R_{c}=\frac{1}{\left|N(c)\right|}\sum_{j\in N(c)}\max\left(0,s_{c,j}{\hat{\varrho }}_{c,j}\right).$$

At least 20 jointly valid one-minute observations are required to calculate a pairwise correlation; otherwise, the corresponding pair is omitted.

A channel-specific ridge regression predictor $g_{c}\left(\cdot \right)$ is additionally fitted using only the training data to predict channel *c* from its related channels. Its normalized cross-channel residual in the current window is

(70) $$E_{c}=\frac{{\mathrm{median}}_{t}\left|x_{t,c}^{h,\mathrm{num}}-g_{c}\left(x_{t,N(c)}^{h,\mathrm{num}}\right)\right|}{\mathrm{MAD}\left(x_{\cdot ,c}^{h,\mathrm{num}}\right)+\varepsilon },\;\varepsilon =10^{-6},$$

where MAD(·) denotes the median absolute deviation. For electrical channels, the physical relation violation rate is calculated using P≈VI:

(71) $$V_{c}=\frac{1}{T_{h}}\sum_{t=1}^{T_{h}}I\left(\frac{|P_{t}-V_{t}I_{t}|}{|P_{t}|+\varepsilon }>\xi_{P}\right),\;\xi_{P}=0.10.$$

For channels without an explicit algebraic relation, $V_{c}$ is set to zero. The cross-channel consistency score is then defined as

(72) $$\chi_{c}=0.5R_{c}+0.3\exp\left(-E_{c}\right)+0.2\left(1-V_{c}\right),\;\chi_{c}\in \left[0,1\right].$$

A larger $\chi_{c}$ indicates that the channel agrees with related sensors, is predictable from their simultaneous states, and does not violate the predefined physical relationships. Equation (72) is calculated directly from the preprocessed observations and the fixed training set predictor; it does not use $\rho_{c}$ or any other reliability weight.

The remaining channel quality descriptors are the missing ratio, prolonged-missingness ratio, local instability, spectral anomaly, and historical distribution shift:

(73) $$m_{c}=1-\frac{1}{T_{h}}\sum_{t=1}^{T_{h}}M_{t,c}^{h},\;g_{c}^{\mathrm{long}}=\frac{1}{T_{h}}\sum_{t=1}^{T_{h}}I\left(\Delta_{t,c}^{h}>G_{\max}\right),$$

(74) $$v_{c}=\frac{\mathrm{MAD}\left(x_{\cdot ,c}^{h,\mathrm{num}}\right)}{{\mathrm{MAD}}_{c}^{\mathrm{train}}+\varepsilon },\;d_{c}=\frac{\left|\mathrm{median}\left(x_{\cdot ,c}^{h,\mathrm{num}}\right)-{\mathrm{median}}_{c}^{\mathrm{train}}\right|}{{\mathrm{MAD}}_{c}^{\mathrm{train}}+\varepsilon }.$$

Let $s_{c}^{\mathrm{spec}}$ denote the proportion of spectral energy outside the channel-specific normal-frequency band estimated from the training data. The complete quality vector is

(75) $$q_{c}={\left[m_{c},\;g_{c}^{\mathrm{long}},\;v_{c},\;s_{c}^{\mathrm{spec}},\;d_{c},\;1-\chi_{c}\right]}^{⊤}\in R^{6}.$$

A non-iterative multilayer perceptron maps $q_{c}$ to a reliability logit:

(76) $$e_{c}^{\left(1\right)}=\phi \left(W_{r}^{\left(1\right)}q_{c}+b_{r}^{\left(1\right)}\right),$$

(77) $$e_{c}^{\left(j\right)}=\phi \left(W_{r}^{\left(j\right)}e_{c}^{\left(j-1\right)}+b_{r}^{\left(j\right)}\right),\;j=2,\ldots ,N_{r},$$

(78) $$a_{c}=w_{r}^{⊤}e_{c}^{\left(N_{r}\right)}+b_{r},\;\rho_{c}=\frac{\exp(a_{c})}{\sum_{v=1}^{C_{h}}\exp\left(a_{v}\right)}.$$

The reliability scores are computed in one forward pass and satisfy $\rho_{c}>0$ and $\sum_{c=1}^{C_{h}}\rho_{c}=1$. No reliability score is used in the construction of $q_{c}$, and no iterative initialization, update, or convergence procedure is employed.

After the channel features are projected into a shared hidden space, the reliability-weighted temporal representation is

(79) $${\tilde{F}}_{t}=\sum_{c=1}^{C_{h}}\rho_{c}B_{c}F_{t,c},$$

where $B_{c}$ maps channel *c* to the shared feature space. If channel *c* is perturbed by $\epsilon_{c}$, the perturbation satisfies

(80) $${∥\sum_{c=1}^{C_{h}}\rho_{c}B_{c}\epsilon_{c}∥}_{2}\leq \sum_{c=1}^{C_{h}}\rho_{c}{∥B_{c}∥}_{2}{∥\epsilon_{c}∥}_{2}.$$

Assuming $∥B_{c}{∥}_{2}\leq L_{B}$ for all channels,

(81) $${∥\Delta {\tilde{F}}_{t}∥}_{2}\leq L_{B}\max_{1\leq c\leq C_{h}}{∥\epsilon_{c}∥}_{2}.$$

Thus, assigning a small reliability weight to an anomalous or inconsistent channel suppresses its influence on the fused hardware representation.

The weighted sequence is subsequently processed by $N_{t}$ temporal Transformer layers. Let $Y^{\left(0\right)}=\tilde{F}$. The temporal correlation matrix in layer *n* is

(82) $$S^{\left(n\right)}=\frac{Y^{\left(n-1\right)}W_{q}^{\left(n\right)}{\left(Y^{\left(n-1\right)}W_{k}^{\left(n\right)}\right)}^{⊤}}{\sqrt{D_{t}}}+B_{\Delta },$$

where $B_{\Delta }$ is generated from the elapsed sampling intervals. The temporal state updates are

(83) $${\tilde{Y}}^{\left(n\right)}=\mathrm{Norm}\left[Y^{\left(n-1\right)}+\mathrm{Softmax}\left(S^{\left(n\right)}\right)Y^{\left(n-1\right)}W_{v}^{\left(n\right)}\right],$$

(84) $$Y^{\left(n\right)}=\mathrm{Norm}\left[{\tilde{Y}}^{\left(n\right)}+{\mathrm{FFN}}_{h}^{\left(n\right)}\left({\tilde{Y}}^{\left(n\right)}\right)\right].$$

After $N_{t}$ layers, the time-step-level state matrix $Y^{\left(N_{t}\right)}\in R^{T_{h}\times D_{t}}$ is retained. Window-level attention pooling is performed as

(85) $$\beta_{\tau }=\frac{\exp\left[v_{h}^{⊤}\tanh\left(W_{h}Y_{\tau }^{\left(N_{t}\right)}\right)\right]}{\sum_{j=1}^{T_{h}}\exp\left[v_{h}^{⊤}\tanh\left(W_{h}Y_{j}^{\left(N_{t}\right)}\right)\right]},$$

(86) $$Z^{h}=\sum_{\tau =1}^{T_{h}}\beta_{\tau }Y_{\tau }^{\left(N_{t}\right)}.$$

The module outputs both the time-step-level hardware features $Y^{\left(N_{t}\right)}$ and the window-level infrastructure pressure representation $Z^{h}$. The former is used by the asynchronous cross-modal interaction module, whereas the latter is used for reliability-aware fusion and market state prediction.

#### 3.3.4. Asynchronous Cross-Modal Fusion and Prediction Module

The asynchronous cross-modal fusion and prediction module receives the event-level semantic sequence $S_{q}\in R^{T_{s}\times D_{s}}$ generated by the semantic encoding module and the hardware state sequence $H_{q}\in R^{T_{h}\times D_{h}}$ generated by the hardware temporal modeling module for prediction window *q*. Here, $T_{s}$ and $T_{h}$ denote the numbers of textual events and hardware time steps, respectively. Given that textual events arrive irregularly while the hardware sequence is sampled at one-minute intervals, the two sequences are not assumed to have position-wise correspondence. Their interaction is modeled through shared space projection, asynchronous soft alignment, bidirectional cross-modal attention, reliability-aware gated fusion, and parallel multitask prediction.

**Figure 3:**
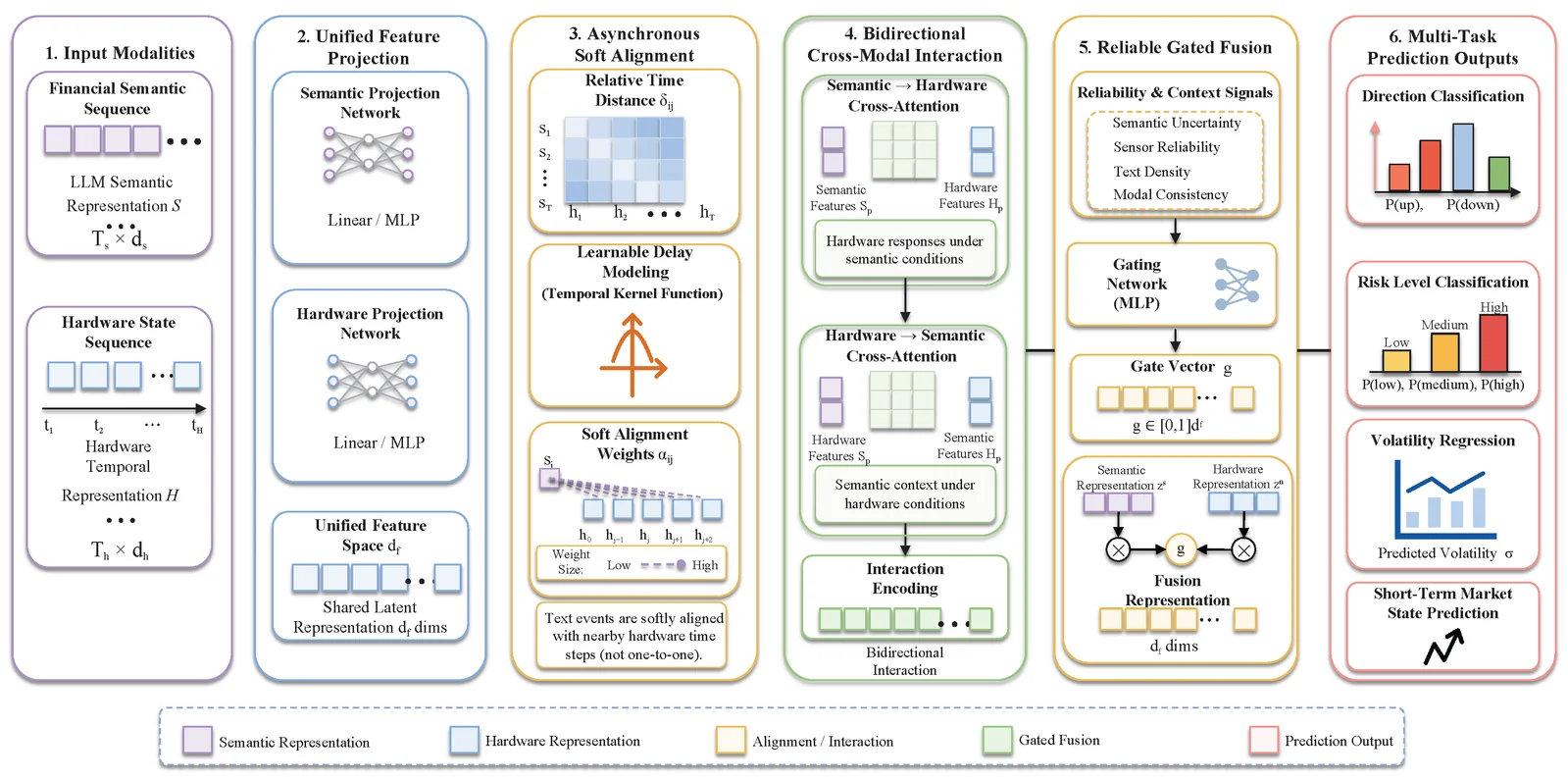
Overall architecture of the asynchronous cross-modal fusion and prediction module in HSF-LLMNet. The gate receives pooled semantic and hardware representations together with window-level semantic uncertainty, overall sensor reliability, text density, cross-modal consistency, and modality-availability indicators.

As shown in Figure 3, two independent projection networks first map the semantic and hardware features into a shared feature space of dimension $D_{f}$:

(87) $${\hat{S}}_{q}=P_{s}\left(S_{q}\right),\;{\hat{H}}_{q}=P_{h}\left(H_{q}\right),$$

where ${\hat{S}}_{q}\in R^{T_{s}\times D_{f}}$ and ${\hat{H}}_{q}\in R^{T_{h}\times D_{f}}$. Each projection network contains two fully connected layers with GELU activation and layer normalization.

Let $\tau_{q,i}$ denote the publication time of semantic event *i*, and let $T_{q,j}^{h}$ denote the timestamp of hardware state *j*. Their signed temporal distance, measured in minutes, is

(88) $$\delta_{q,ij}=T_{q,j}^{h}-\tau_{q,i}.$$

The semantic-to-hardware alignment score is defined as

(89) $$e_{q,ij}^{s\to h}=\frac{\left({\hat{s}}_{q,i}W_{q}^{s}\right){\left({\hat{h}}_{q,j}W_{k}^{h}\right)}^{⊤}}{\sqrt{D_{a}}}-\gamma \left|\delta_{q,ij}\right|+w_{\delta }^{⊤}\psi \left(\delta_{q,ij}\right),$$

where γ≥0 controls absolute temporal decay and $\psi \left(\delta_{q,ij}\right)\in R^{D_{\delta }}$ is produced by a two-layer temporal embedding network. The corresponding alignment weights and hardware context vector are

(90) $$a_{q,ij}^{s\to h}=\frac{\exp\left(e_{q,ij}^{s\to h}\right)}{\sum_{r=1}^{T_{h}}\exp\left(e_{q,ir}^{s\to h}\right)},$$

(91) $$c_{q,i}^{s\to h}=\sum_{j=1}^{T_{h}}a_{q,ij}^{s\to h}{\hat{h}}_{q,j}.$$

The reverse branch uses independent parameters because the relation between an event and a subsequent hardware response is not assumed to be symmetric. Its alignment score is

(92) $$e_{q,ji}^{h\to s}=\frac{\left({\hat{h}}_{q,j}U_{q}^{h}\right){\left({\hat{s}}_{q,i}U_{k}^{s}\right)}^{⊤}}{\sqrt{D_{a}}}+u_{\delta }^{⊤}\psi \left(-\delta_{q,ij}\right),$$

and the reverse alignment weights and semantic context vector are

(93) $$b_{q,ji}^{h\to s}=\frac{\exp\left(e_{q,ji}^{h\to s}\right)}{\sum_{r=1}^{T_{s}}\exp\left(e_{q,jr}^{h\to s}\right)},$$

(94) $$c_{q,j}^{h\to s}=\sum_{i=1}^{T_{s}}b_{q,ji}^{h\to s}{\hat{s}}_{q,i}.$$

The aligned context vectors are combined with their original representations through two independent interaction encoders:

(95) $$r_{q,i}^{s}=F_{s}\left({\hat{s}}_{q,i}∥c_{q,i}^{s\to h}∥{\hat{s}}_{q,i}⊙c_{q,i}^{s\to h}∥\left|{\hat{s}}_{q,i}-c_{q,i}^{s\to h}\right|\right),$$

(96) $$r_{q,j}^{h}=F_{h}\left({\hat{h}}_{q,j}∥c_{q,j}^{h\to s}∥{\hat{h}}_{q,j}⊙c_{q,j}^{h\to s}∥\left|{\hat{h}}_{q,j}-c_{q,j}^{h\to s}\right|\right).$$

Both interaction encoders output vectors in $R^{D_{f}}$.

Attention pooling is applied separately to the semantic and hardware interaction sequences:

(97) $$\pi_{q,i}^{s}=\frac{\exp\left[v_{s}^{⊤}\tanh\left(W_{s}r_{q,i}^{s}\right)\right]}{\sum_{k=1}^{T_{s}}\exp\left[v_{s}^{⊤}\tanh\left(W_{s}r_{q,k}^{s}\right)\right]},\;{\bar{r}}_{q}^{s}=\sum_{i=1}^{T_{s}}\pi_{q,i}^{s}r_{q,i}^{s},$$

(98) $$\pi_{q,j}^{h}=\frac{\exp\left[v_{h}^{⊤}\tanh\left(W_{h}r_{q,j}^{h}\right)\right]}{\sum_{k=1}^{T_{h}}\exp\left[v_{h}^{⊤}\tanh\left(W_{h}r_{q,k}^{h}\right)\right]},\;{\bar{r}}_{q}^{h}=\sum_{j=1}^{T_{h}}\pi_{q,j}^{h}r_{q,j}^{h}.$$

The reliability and context signals supplied to the gating network are listed in Table 4. All of these quantities are computed before the gate and therefore do not depend on the gate output.

The window-level semantic uncertainty is calculated from the calibrated event-level values $u_{q,i}$:

(99) $${\bar{u}}_{q}=\left\{\begin{array}{cc} \sum_{i=1}^{T_{s}}\pi_{q,i}^{s}u_{q,i},\; & T_{s}>0,\\ 1,\; & T_{s}=0. \end{array}\right.$$

Thus, ${\bar{u}}_{q}\in \left[0,1\right]$, and a larger value indicates that the semantic information within the current prediction window is less reliable.

For channel *c*, let $a_{q,c}$ denote the reliability logit generated by the channel-quality MLP in the hardware module. In addition to the normalized channel weight used for hardware aggregation, an absolute reliability score is retained:

(100) $${\tilde{\rho }}_{q,c}=\mathrm{Sigmoid}\left(a_{q,c}\right).$$

Let

(101) $$\chi_{q,c}=\frac{1}{T_{h}}\sum_{j=1}^{T_{h}}M_{q,j,c}^{h}$$

denote the valid observation coverage of channel *c*. The window-level sensor reliability is

(102) $${\bar{\rho }}_{q}=\frac{\sum_{c=1}^{C_{h}}\chi_{q,c}{\tilde{\rho }}_{q,c}}{\sum_{c=1}^{C_{h}}\chi_{q,c}+\varepsilon },\;\varepsilon =10^{-8}.$$

Unlike the normalized channel weights, which sum to one, ${\bar{\rho }}_{q}$ represents absolute hardware quality and can decrease when all channels are missing, unstable, or inconsistent.

The normalized text density is

(103) $$n_{q}=\min\left(\frac{T_{s}}{N_{d}},1\right),\;N_{d}=64.$$

The modality availability indicators are

(104) $$m_{q}^{s}=I\left(T_{s}>0\right),\;m_{q}^{h}=I\left(\frac{1}{T_{h}C_{h}}\sum_{j=1}^{T_{h}}\sum_{c=1}^{C_{h}}M_{q,j,c}^{h}\geq 0.80\right).$$

Cross-modal consistency is computed from two quantities that are available before gated fusion. First, the cosine agreement between the pooled representations is

(105) $$c_{q}^{\cos}=\frac{1}{2}\left[1+\frac{{\left(W_{c}^{s}{\bar{r}}_{q}^{s}\right)}^{⊤}\left(W_{c}^{h}{\bar{r}}_{q}^{h}\right)}{{∥W_{c}^{s}{\bar{r}}_{q}^{s}∥}_{2}{∥W_{c}^{h}{\bar{r}}_{q}^{h}∥}_{2}+\varepsilon }\right],$$

where $c_{q}^{\cos}\in \left[0,1\right]$.

Second, the agreement between the two directional alignment distributions is measured. Define the two joint event–hardware distributions as

(106) $$P_{q,ij}=\pi_{q,i}^{s}a_{q,ij}^{s\to h},\;Q_{q,ij}=\pi_{q,j}^{h}b_{q,ji}^{h\to s}.$$

Both distributions satisfy $\sum_{i=1}^{T_{s}}\sum_{j=1}^{T_{h}}P_{q,ij}=1$ and $\sum_{i=1}^{T_{s}}\sum_{j=1}^{T_{h}}Q_{q,ij}=1$. Let

(107) $$M_{q}^{PQ}=\frac{1}{2}\left(P_{q}+Q_{q}\right).$$

The attention consistency score is defined through the Jensen–Shannon divergence:

(108) $$c_{q}^{\mathrm{att}}=1-\frac{\frac{1}{2}D_{\mathrm{KL}}\left(P_{q}∥M_{q}^{PQ}\right)+\frac{1}{2}D_{\mathrm{KL}}\left(Q_{q}∥M_{q}^{PQ}\right)}{\log2},$$

where $c_{q}^{\mathrm{att}}\in \left[0,1\right]$. The complete cross-modal consistency signal is

(109) $$c_{q}=\left\{\begin{array}{cc} \alpha_{c}c_{q}^{\cos}+\left(1-\alpha_{c}\right)c_{q}^{\mathrm{att}},\; & m_{q}^{s}=1,\;m_{q}^{h}=1,\\ 0,\; & \mathrm{otherwise}, \end{array}\right.\;\alpha_{c}=0.5.$$

Therefore, $c_{q}$ is high only when the pooled semantic and hardware representations are similar and the two alignment directions assign compatible event–hardware associations. It is computed directly from the aligned representations and attention matrices and does not depend on the gate vector.

The gate contains two fully connected layers with hidden dimension $D_{g}=128$ and dropout rate 0.10. Its hidden representation is

(110) $$\begin{array}{cc} h_{q}^{g}=\mathrm{GELU}(\; & W_{s}^{g}{\bar{r}}_{q}^{s}+W_{h}^{g}{\bar{r}}_{q}^{h}+w_{u}^{g}{\bar{u}}_{q}+w_{\rho }^{g}{\bar{\rho }}_{q}\\ \; & +w_{n}^{g}n_{q}+w_{c}^{g}c_{q}+w_{ms}^{g}m_{q}^{s}+w_{mh}^{g}m_{q}^{h}+b_{g}^{\left(1\right)}), \end{array}$$

where $W_{s}^{g},W_{h}^{g}\in R^{D_{g}\times D_{f}}$ and $w_{u}^{g},w_{\rho }^{g},w_{n}^{g},w_{c}^{g},w_{ms}^{g},w_{mh}^{g}\in R^{D_{g}}$. Consequently, each scalar reliability signal is multiplied by a learned $D_{g}$-dimensional coefficient vector and is expanded through learned broadcasting rather than being manually repeated.

The feature-wise gate is

(111) $$g_{q}=\mathrm{Sigmoid}\left[W_{g}^{\left(2\right)}\mathrm{Dropout}\left(h_{q}^{g},0.10\right)+b_{g}^{\left(2\right)}\right],\;g_{q}\in {\left(0,1\right)}^{D_{f}}.$$

The output layer maps the $D_{g}$-dimensional hidden representation to $D_{f}$ values, so one gate coefficient is produced for each fused feature dimension.

To handle a completely unavailable modality, the gate is adjusted as

(112) $${\tilde{g}}_{q}=m_{q}^{s}\left[m_{q}^{h}g_{q}+\left(1-m_{q}^{h}\right)1\right].$$

When both modalities are available, ${\tilde{g}}_{q}=g_{q}$. When only semantic information is available, ${\tilde{g}}_{q}=1$; when only hardware information is available, ${\tilde{g}}_{q}=0$. Samples for which both modalities are unavailable are excluded during data alignment.

The fused representation is

(113) $$z_{q}^{f}={\tilde{g}}_{q}⊙{\bar{r}}_{q}^{s}+\left(1-{\tilde{g}}_{q}\right)⊙{\bar{r}}_{q}^{h}.$$

For each feature dimension *k*, when both modalities are present,

(114) $$z_{q,k}^{f}=g_{q,k}{\bar{r}}_{q,k}^{s}+\left(1-g_{q,k}\right){\bar{r}}_{q,k}^{h},$$

and therefore

(115) $$\min\left({\bar{r}}_{q,k}^{s},{\bar{r}}_{q,k}^{h}\right)\leq z_{q,k}^{f}\leq \max\left({\bar{r}}_{q,k}^{s},{\bar{r}}_{q,k}^{h}\right).$$

The gating regularization is applied only to samples for which both modalities are available. Let $B_{\mathrm{both}}$ denote this subset of a training mini-batch and let

(116) $${\bar{g}}_{B}=\frac{1}{|B_{\mathrm{both}}|}\sum_{q\in B_{\mathrm{both}}}g_{q}.$$

The regularization term is

(117) $$\begin{array}{cc} L_{\mathrm{gate}}=\; & \frac{1}{D_{f}}{∥{\bar{g}}_{B}-\frac{1}{2}1∥}_{2}^{2}\\ \; & -\frac{\kappa_{g}}{|B_{\mathrm{both}}|D_{f}}\sum_{q\in B_{\mathrm{both}}}\sum_{k=1}^{D_{f}}H_{\mathrm{bin}}\left(g_{q,k}\right),\;\kappa_{g}=0.10, \end{array}$$

where

(118) $$H_{\mathrm{bin}}\left(g\right)=-g\log\left(g+\varepsilon \right)-\left(1-g\right)\log\left(1-g+\varepsilon \right).$$

The first term prevents the batch-average gate from remaining close to zero or one across all feature dimensions, while the entropy term discourages premature saturation of individual gates. Since the regularization is omitted when one modality is unavailable, it does not prevent deterministic selection of the remaining valid modality.

Finally, three independent output heads operate on the same fused representation:

(119) $${\hat{y}}_{q}^{\mathrm{dir}}=\mathrm{Softmax}\left[O_{\mathrm{dir}}\left(z_{q}^{f}\right)\right],$$

(120) $${\hat{y}}_{q}^{\mathrm{risk}}=\mathrm{Softmax}\left[O_{\mathrm{risk}}\left(z_{q}^{f}\right)\right],$$

(121) $${\hat{y}}_{q}^{\mathrm{vol}}=\mathrm{Softplus}\left[O_{\mathrm{vol}}\left(z_{q}^{f}\right)\right].$$

The direction head produces two probabilities, the risk head produces the probabilities of low, medium, and high risk, and the volatility head produces a nonnegative realized-volatility estimate. These are parallel outputs rather than nested predictions.

The complete multitask objective is

(122) $$L=\lambda_{d}L_{\mathrm{dir}}+\lambda_{r}L_{\mathrm{risk}}+\lambda_{v}L_{\mathrm{vol}}+\lambda_{a}L_{\mathrm{align}}+\lambda_{g}L_{\mathrm{gate}},$$

where $L_{\mathrm{dir}}$ is binary cross-entropy, $L_{\mathrm{risk}}$ is class-weighted cross-entropy, and $L_{\mathrm{vol}}$ is the Huber regression loss. The bidirectional alignment regularization is

(123) $$L_{\mathrm{align}}=\frac{1}{|B_{\mathrm{both}}|}\sum_{q\in B_{\mathrm{both}}}D_{\mathrm{JS}}\left(P_{q}∥Q_{q}\right),$$

which encourages the semantic-to-hardware and hardware-to-semantic branches to produce compatible associations without forcing them to share parameters.

The revised formulation specifies every reliability and context signal entering the gate. Semantic uncertainty ${\bar{u}}_{q}$, sensor reliability ${\bar{\rho }}_{q}$, text density $n_{q}$, cross-modal consistency $c_{q}$, and the two availability indicators are scalars, while ${\bar{r}}_{q}^{s}$ and ${\bar{r}}_{q}^{h}$ are $D_{f}$-dimensional vectors. The scalar signals are expanded by learned coefficient vectors within Equation (110), and Equation (111) produces a $D_{f}$-dimensional gate. Cross-modal consistency is computed before the gate from shared-space cosine similarity and bidirectional alignment agreement, eliminating the undefined “Reliability & Context Signals” description in the original formulation.

## 4. Results and Discussion

### 4.1. Experimental Configuration

#### 4.1.1. Hardware and Software Platform

The experimental platform consisted of a model-training server, hardware sensor acquisition devices, and an edge inference node. Model training was performed on a server equipped with an Intel Xeon Silver 4314 processor, 128 GB DDR4 memory, an NVIDIA GeForce RTX 4090 GPU with 24 GB video memory, and a 4-TB NVMe solid-state drive. The trading infrastructure used for sensor acquisition included Dell PowerEdge R750 computing nodes and an NVIDIA Jetson AGX Xavier edge node. Electrical variables were measured using INA219 and INA226 current and voltage monitoring modules, rack temperature and relative humidity were measured using Sensirion SHT35 sensors, and equipment vibration was recorded using MPU6050 three-axis inertial sensors. Network latency, throughput, packet loss rate, and port traffic were obtained from Cisco Catalyst C9300-24T switches and network probes. Edge inference was evaluated on an NVIDIA Jetson AGX Orin 64-GB Developer Kit operating in MAXN mode to assess the feasibility of local short-term prediction and risk warning.

The training server ran Ubuntu 20.04.6 LTS with Python 3.10, PyTorch 2.3, CUDA 12.1, and cuDNN 8.9. Text processing, semantic encoding, and event extraction were implemented using Transformers 4.37, jieba 0.42, NLTK 3.8, and scikit-learn 1.3. Corpus cleaning, deduplication, timestamp normalization, and asset matching were implemented using Pandas 2.1, NumPy 1.26, and regular expressions. Hardware preprocessing, including anomaly screening, missing-value completion, one-minute aggregation, local detrending, and standardization, was implemented using Pandas, NumPy, SciPy 1.11, and scikit-learn. ONNX Runtime 1.16 and TensorRT 10.0 were used for edge-inference acceleration. The deployment model was exported using ONNX opset 17 and compiled using TensorRT with FP16 precision. Matplotlib 3.8 and SciPy were used for statistical analysis and result visualization.

The dataset was divided chronologically according to the prediction origin $T_{q}$. The future price $P_{T_{q}+\Delta T}$ was used only to construct the supervised label and was never included in the model input, preprocessing statistics, feature selection, or model-selection procedure. To prevent samples near a partition boundary from using future prices belonging to the subsequent partition, a sample was retained only when its complete target interval ended within the same training, validation, or test portion. Since the maximum evaluated prediction horizon was 60 min, a 60 min purge interval was applied at each partition boundary. A 24 h embargo was additionally introduced to prevent textual look-back windows from overlapping across adjacent portions. After removing samples within the purge and embargo intervals, the earliest 70% of the eligible prediction origins were used for training, the following 15% for validation, and the latest 15% for testing. Five-fold evaluation followed a rolling-origin expanding-window design, in which the training interval expanded over time and every validation and test interval occurred strictly later than its corresponding training interval. All normalization parameters, anomaly thresholds, missing-value statistics, local baseline parameters, feature selection settings, label thresholds, and uncertainty calibration mappings were fitted exclusively on the training portion of each split and then kept fixed during validation and testing. The validation portion was used for hyperparameter selection, early stopping, and checkpoint selection, whereas the test portion was accessed only for final evaluation. Data augmentation was also restricted to the training portion.

The principal prediction horizon was 30 min, and an additional 60 min horizon was used for comparison. The textual look-back window was set to $L_{d}=24$ h, and the hardware look-back window was set to $L_{x}=60$ min. All sensor streams were aggregated into non-overlapping one-minute intervals. For each sensor channel, the mean, standard deviation, minimum, maximum, least-squares slope, and mean absolute difference between consecutive native observations were retained within each interval. The original level, causal baseline, detrended residual, and their separate temporal changes were also preserved. The model therefore received 60 min-level hardware vectors for each prediction window. Raw native-frequency sensor segments were not supplied through an additional model branch.

The cached LLM-derived semantic representation had a dimension of 768, whereas the hardware temporal representation and the shared cross-modal representation had dimensions of 256. The hardware branch consisted of three gated causal-convolution layers followed by $N_{t}=2$ temporal Transformer encoding layers with a hidden dimension of 256, eight attention heads, a feed-forward dimension of 1024, and a dropout rate of 0.3. The bidirectional cross-attention module used four attention heads, and the shared cross-modal dimension was set to $D_{f}=256$. The complete trainable fusion network contained 22.14 million parameters, including the semantic projection layers, hardware temporal encoder, asynchronous bidirectional interaction module, reliability-aware gate, and three multitask prediction heads.

Fusion-network training used AdamW with an initial learning rate of $1\times 10^{-4}$, a weight decay of $1\times 10^{-5}$, a mini-batch size of 64, and a maximum of 100 epochs. Training was performed using bfloat16 automatic mixed precision, and the gradient norm was clipped at 1.0. Training was stopped when the validation loss did not decrease for 10 consecutive epochs. The multitask loss weights were set to $\lambda_{c}=1.0$, $\lambda_{v}=0.8$, $\lambda_{r}=0.8$, $\lambda_{u}=0.3$, and $\lambda_{s}=0.5$. The probability of synonym-based text rewriting was 0.15, and texts with calibrated semantic uncertainty greater than 0.70 were excluded from automatic augmentation. The sensor channel dropout probability was p=0.10, and Gaussian noise was generated with a standard deviation equal to 0.05 times the training set standard deviation of the corresponding sensor feature. Temporal perturbation was restricted to ±2 one-minute intervals and was not permitted to move any observation beyond the prediction origin $T_{q}$.

#### 4.1.2. Baseline Models and Evaluation Metrics

Logistic Regression [46], XGBoost [47], LightGBM [48], LSTM [49], Transformer [50], Informer [51], Text-only BERT/FinBERT [52], LLM-text-only [53], Sensor-only Transformer [54], and a conventional feature-concatenation fusion model were selected as baseline methods. Logistic regression performs market direction or risk state classification using a linear decision boundary and is characterized by structural simplicity and strong interpretability. XGBoost iteratively minimizes prediction error through gradient-boosted decision trees and is well suited to structured features such as prices, trading volumes, and technical indicators. LightGBM improves computational efficiency through an optimized gradient-boosting framework and is suitable for large-scale feature modeling. LSTM captures long-term dependencies in market sequences through gated recurrent mechanisms and can model lagged relationships among prices and trading volumes. Transformer captures global dependencies across different time steps through self-attention and can emphasize critical moments associated with market shocks. Informer reduces the computational cost of attention for long-sequence forecasting and is therefore suitable for trend modeling over extended temporal windows. Text-only BERT/FinBERT relies exclusively on texts for semantic sentiment recognition and can capture sentiment polarity and event-related information from corpora. LLM-text-only performs prediction solely using semantic representations generated by a large language model and can extract richer features related to event impact and semantic uncertainty. Sensor-only Transformer uses only hardware sensor time-series data to characterize system operating states and is employed to examine the independent contribution of hardware signals to market pressure perception. The conventional feature-concatenation fusion model directly concatenates textual, sensor, and market features before prediction and serves as a simple multimodal fusion reference. Comparisons with these baselines enable the effectiveness of the LLM-based semantic sentiment encoder, the reliable hardware sensor modeling mechanism, and the asynchronous cross-modal fusion mechanism in HSF-LLMNet to be systematically evaluated for short-term market volatility prediction.

The evaluation metrics included accuracy, precision, recall, F1-score, area under the receiver operating characteristic curve (AUC), Matthews correlation coefficient (MCC), mean absolute error (MAE), root mean squared error (RMSE), epsilon-stabilized mean absolute percentage error (MAPE), normalized MAE (NMAE), mean absolute scaled error (MASE), $R^{2}$, effective warning time (EWT), false alarm rate (FAR), and missed alarm rate (MAR). Classification metrics were used for market direction prediction, regression metrics were used for realized volatility prediction, and EWT, FAR, and MAR were calculated at the event level according to the warning protocol defined below.

(124) $$\mathrm{Accuracy}=\frac{TP+TN}{TP+TN+FP+FN},\;\mathrm{Precision}=\frac{TP}{TP+FP},\;\mathrm{Recall}=\frac{TP}{TP+FN},$$

(125) $$F1=\frac{2\;\mathrm{Precision}\;\mathrm{Recall}}{\mathrm{Precision}+\mathrm{Recall}},\;\mathrm{MCC}=\frac{TP\times TN-FP\times FN}{\sqrt{\left(TP+FP)(TP+FN)(TN+FP)(TN+FN\right)}},$$

(126) $$\mathrm{AUC}=P\left(s^{+}>s^{-}\right),\;\mathrm{MAE}=\frac{1}{n}\sum_{i=1}^{n}\left|y_{i}-{\hat{y}}_{i}\right|,\;\mathrm{RMSE}=\sqrt{\frac{1}{n}\sum_{i=1}^{n}{\left(y_{i}-{\hat{y}}_{i}\right)}^{2}},$$

(127) $${\mathrm{MAPE}}_{\epsilon }=\frac{100}{n}\sum_{i=1}^{n}\frac{|y_{i}-{\hat{y}}_{i}|}{\max(|y_{i}|,\epsilon )},\;\epsilon =10^{-3},$$

(128) $$\mathrm{NMAE}=\frac{\mathrm{MAE}}{\frac{1}{n}\sum_{i=1}^{n}\left|y_{i}\right|},\;\mathrm{MASE}=\frac{\frac{1}{n}\sum_{i=1}^{n}\left|y_{i}-{\hat{y}}_{i}\right|}{\frac{1}{n}\sum_{i=1}^{n}\left|y_{i}-{\tilde{y}}_{i}\right|},$$

(129) $$R^{2}=1-\frac{\sum_{i=1}^{n}{\left(y_{i}-{\hat{y}}_{i}\right)}^{2}}{\sum_{i=1}^{n}{\left(y_{i}-\bar{y}\right)}^{2}}.$$

Here, TP, TN, FP, and FN denote the numbers of true-positive, true-negative, false-positive, and false-negative direction predictions, respectively. The variables $s^{+}$ and $s^{-}$ denote the prediction scores for positive and negative direction samples. The variables $y_{i}$, ${\hat{y}}_{i}$, and $\bar{y}$ denote the realized-volatility target, its prediction, and the mean target value, respectively. The naive forecast ${\tilde{y}}_{i}$ used in MASE was the realized volatility observed during the immediately preceding 30 min interval for the same index. The primary regression metrics were micro-averaged over all test windows. Macro-averaged results were additionally calculated separately for each of the eight indices and then averaged with equal index weights.

Realized volatility was positive but could be close to zero. In the pooled out-of-sample test data, the minimum realized-volatility target was $2.1\times 10^{-4}$, no target was exactly zero, and 188 of 25,381 observations, corresponding to 0.74%, were below $10^{-3}$. The value $\epsilon =10^{-3}$ was fixed before test set evaluation. When $y_{i}<\epsilon$, the MAPE denominator was truncated to ϵ rather than deleting the observation. Since MAPE remains sensitive to this cutoff, it was retained as an auxiliary metric, whereas NMAE and MASE were used as scale-robust measures. The pooled test set mean realized volatility was 0.0463; therefore, an MAE of 0.0089 corresponded to an NMAE of 0.192. The corresponding MASE was 0.684, indicating a lower absolute error than the previous window volatility benchmark.

For risk warning, the risk head produced three softmax probabilities, $p_{q}^{L}$, $p_{q}^{M}$, and $p_{q}^{H}$, corresponding to low-, medium-, and high-risk states. The predicted high-risk probability $p_{q}^{H}$ was used as the warning score. For each time-series fold, the alarm threshold was selected exclusively from the validation portion by maximizing event-level F1-score subject to FAR≤10%. The five validation-selected thresholds were 0.59, 0.61, 0.63, 0.64, and 0.68, with a mean of 0.63. No threshold was adjusted using the test data.

A high-risk event began at the first prediction origin for which the future 30 min target entered the high-risk state defined by realized volatility, absolute return, and liquidity stress. Consecutive high-risk windows separated by less than 30 min were merged into one event episode, and the beginning of the first window was defined as $t_{\mathrm{event}}$. An alarm episode began when $p_{q}^{H}$ crossed the validation-selected threshold from below. Repeated threshold exceedances separated by no more than 5 min were merged, and the first crossing was defined as $t_{\mathrm{alarm}}$.

An alarm was eligible for matching when it occurred between 30 and 1 min before the onset of a high-risk event. When several eligible alarms preceded the same event, only the earliest alarm was retained as a true alarm, and the remaining alarms were removed as duplicates. Each alarm could be matched to at most one event. An alarm without a matched event was counted as a false alarm. An alarm generated at or after $t_{\mathrm{event}}$ was treated as a late alarm and was not counted as a successful warning. A high-risk event without an eligible preceding alarm was counted as a missed alarm.

The pooled test data contained 25,381 prediction windows, including 15,842 low-risk windows (62.42%), 6263 medium-risk windows (24.68%), and 3276 high-risk windows (12.90%). After temporally adjacent high-risk windows were merged, 430 distinct high-risk event episodes remained. These event episodes, rather than individual high-risk windows, were used as the units for calculating MAR and EWT.

Let TA denote the number of successfully matched alarm–event pairs, FA the number of unmatched alarm episodes, and MA the number of high-risk events without a matched alarm. The event-level warning metrics were defined as

(130) $${\mathrm{EWT}}_{j}=t_{\mathrm{event},j}-t_{\mathrm{alarm},j},\;\bar{\mathrm{EWT}}=\frac{1}{TA}\sum_{j=1}^{TA}{\mathrm{EWT}}_{j},$$

(131) $$\mathrm{FAR}=\frac{FA}{FA+TA},\;\mathrm{MAR}=\frac{MA}{MA+TA}.$$

EWT was calculated only for successfully matched true alarms. Its mean value was 15.37 min, its median was 14.0 min, and its interquartile range was 9–22 min. The 5th–95th percentile interval was 3–29 min. FAR used the total number of distinct alarm episodes as its denominator, whereas MAR used the total number of merged high-risk events. This event-level protocol prevented repeated predictions within the same risk episode from inflating the warning results.

### 4.2. Market Direction Prediction

This experiment was designed to compare the capability of different methods in predicting market direction over the subsequent 30 min and to examine the contributions of text, hardware sensor information, and asynchronous cross-modal fusion to short-term directional identification.

**Figure 4:**
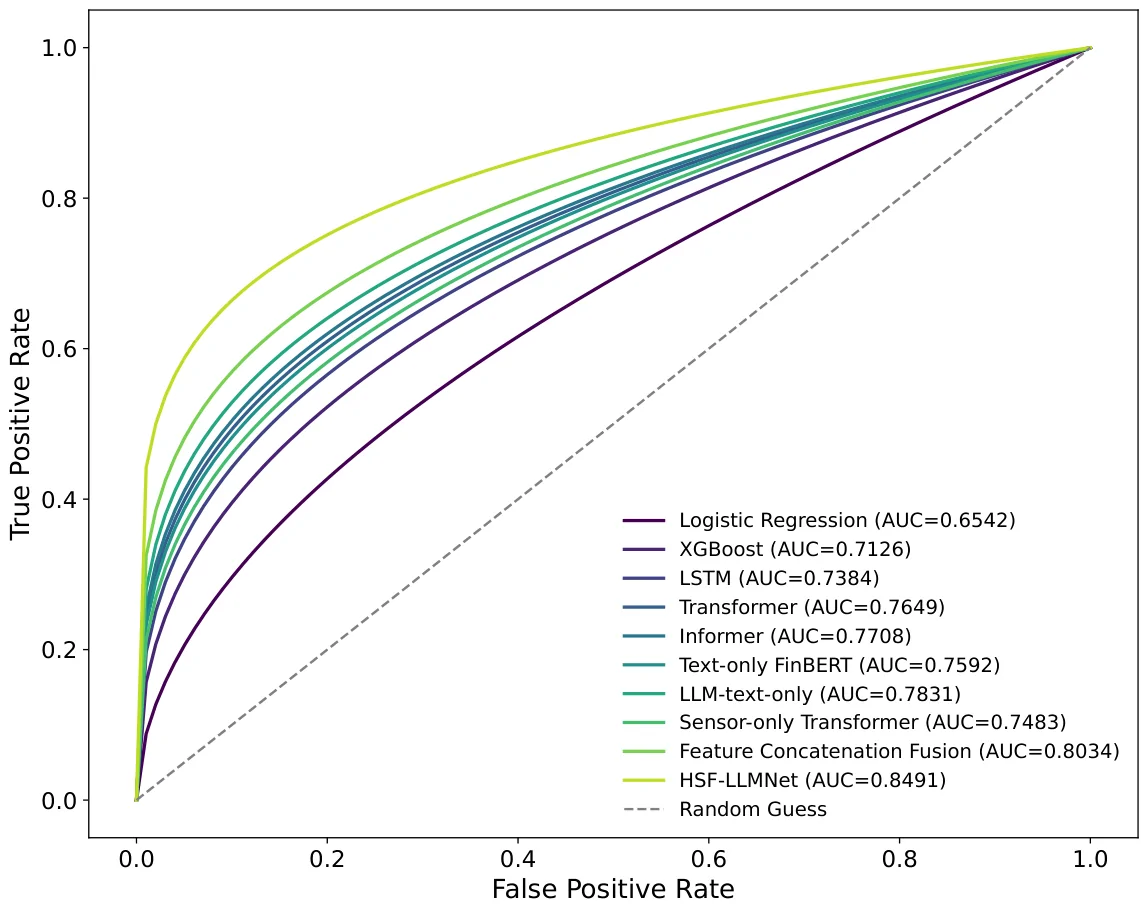
ROC curve comparison of different models for market direction prediction over the subsequent 30 min, with HSF-LLMNet achieving the highest AUC and demonstrating superior class discrimination capability.

As shown in Table 5 and Figure 4, logistic regression achieved an Accuracy of only 61.28% and an MCC of 22.36%, indicating that a linear decision boundary is insufficient for characterizing the nonlinear coupling among market variables. XGBoost increased the Accuracy to 66.35% and the AUC to 71.26%, demonstrating that tree-based models can exploit nonlinear interactions among prices, trading volumes, and technical indicators, although their ability to model continuous temporal dependencies remains limited. Long short term memory (LSTM) achieved an Accuracy of 68.45% and an F1-score of 67.63%. Its gated recurrent structure allows historical states to be retained, resulting in superior performance compared with static models. Transformer and Informer achieved Accuracies of 70.86% and 71.34%, respectively, while the AUC of Informer reached 77.08%. These results indicate that global attention and sparse long-sequence modeling facilitate the identification of critical time points and long-term market trends. Text-only FinBERT achieved an Accuracy of 70.13%, suggesting that domain-specific pretrained textual representations can effectively capture sentiment and event information. LLM-text-only further increased the Accuracy to 72.46% and the MCC to 45.16%, demonstrating that large language models provide stronger representations of event semantics, risk intensity, and contextual relationships. Sensor-only Transformer achieved an Accuracy of 69.28%, confirming that hardware workload, power consumption, and network states possess independent predictive value for market pressure. Feature concatenation fusion achieved an Accuracy of 74.39% and an AUC of 80.34%, indicating that complementary information from multiple sources can substantially improve directional prediction.

HSF-LLMNet achieved the best results across all evaluation metrics, with Accuracy, F1-score, AUC, and MCC values of 78.62%, 77.98%, 84.91%, and 57.36%, respectively. Compared with the second-best fusion model, improvements of 4.23, 4.30, 4.57, and 8.45 percentage points were obtained for these four metrics, respectively. The narrower confidence intervals further indicate greater stability across temporal subsets. From the perspective of model structure, conventional linear models can only learn fixed weighted relationships, while tree-based models depend on local feature partitioning. LSTM models temporal dependencies through recurrent state updates, but long-range information may gradually decay. Although Transformer-based models can establish global relationships through attention, differences in temporal scale and reliability across modalities are not explicitly considered. Direct feature concatenation maps textual and sensor features into the same space without addressing the irregular arrival of news events, delayed hardware responses, or noise caused by abnormal sensors. In contrast, HSF-LLMNet employs an LLM to extract fine-grained event semantics, suppresses missing, drifting, and abnormal channels through sensor reliability weighting, performs asynchronous soft alignment through a learnable temporal kernel, and dynamically adjusts the contributions of the two modalities through bidirectional interaction and channel-wise gating. Consequently, temporal misalignment, noise propagation, and single-modality bias are jointly reduced, producing substantial improvements in Accuracy, AUC, and MCC.

### 4.3. Volatility Prediction and Risk Warning

This experiment was designed to jointly evaluate the continuous prediction capability of different methods for market volatility over the subsequent 30 min and their capability to provide early warnings of risk events. The contributions of text, hardware sensor information, and cross-modal fusion to error control, warning lead time, and alert reliability were also examined.

**Figure 5:**
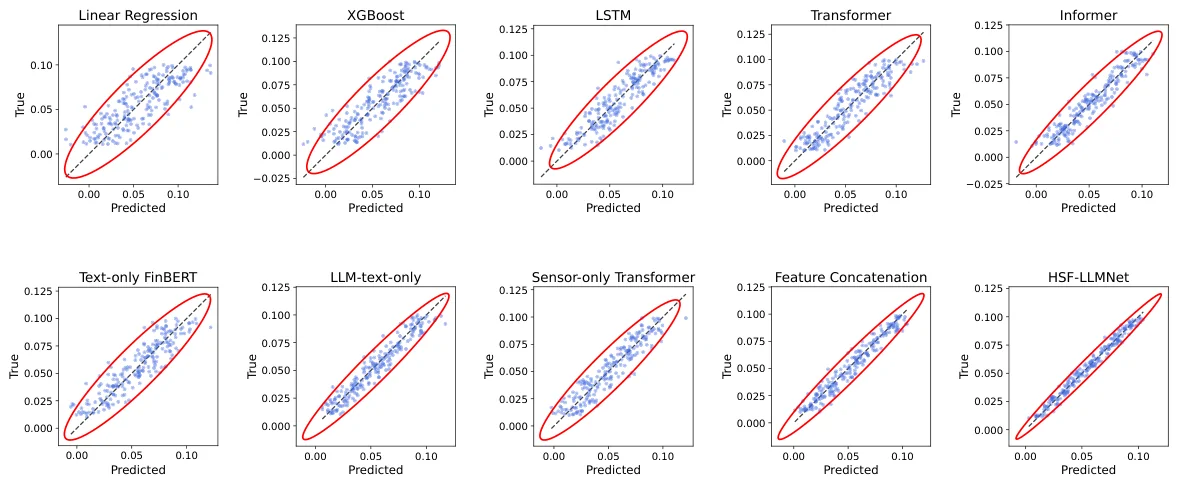
Comparison between predicted and observed volatility values for different models over the subsequent 30 min, where HSF-LLMNet exhibits the closest concentration around the ideal reference line and the highest regression accuracy and consistency.

As shown in Table 6 and Figure 5, logistic/linear regression exhibited the weakest performance, with MAE and RMSE values of 0.0187 and 0.0264, respectively. Its coefficient of determination was only 0.412, the warning time was 6.42 min, and the FAR and MAR reached 18.73% and 22.46%, respectively. These results indicate that linear relationships are insufficient for representing the non-stationarity of volatility. XGBoost reduced the MAE to 0.0152 and increased the coefficient of determination to 0.548, demonstrating that nonlinear threshold relationships can be effectively captured by tree-based models. LSTM further reduced the RMSE to 0.0202 and extended the warning time to 9.27 min, reflecting the ability of recurrent states to model historical dependencies. The coefficients of determination obtained by Transformer and Informer reached 0.657 and 0.671, respectively, while their FAR values decreased to 11.63% and 11.29%. These findings indicate that global attention and sparse long-sequence modeling are beneficial for capturing persistent risk patterns. Text-only FinBERT achieved an MAE of 0.0134, while LLM-text-only further reduced the MAE to 0.0121 and extended the warning time to 11.26 min, demonstrating that large language models can extract more fine-grained event impacts. Sensor-only Transformer achieved a coefficient of determination of 0.641, confirming that hardware workload, power consumption, and network status possess independent early-warning value. Feature concatenation fusion achieved the second-best results, with an MAE of 0.0108 and a warning time of 12.48 min, confirming the pronounced complementarity of multisource information.

HSF-LLMNet achieved the best performance across all evaluation metrics. The MAE, RMSE, and MAPE were reduced to 0.0089, 0.0135, and 9.21%, respectively, while the coefficient of determination increased to 0.812. The average effective warning time reached 15.37 min, and the FAR and MAR were reduced to 6.82% and 8.14%, respectively. From the perspective of model structure, linear regression can only fit fixed-coefficient relationships, whereas tree-based models rely on piecewise partitioning and cannot continuously represent long-term dynamics. LSTM accumulates historical information through recurrent states, but long-range dependencies may gradually decay. Although Transformer and Informer possess global modeling capabilities, the asynchronous relationship between textual events and hardware responses is not explicitly addressed. A text-only model cannot perceive infrastructure pressure, whereas a sensor-only model lacks semantic explanations of market events. Direct concatenation is additionally vulnerable to temporal misalignment and noise propagation. HSF-LLMNet suppresses ambiguous textual information through semantic uncertainty constraints, down-weights abnormal hardware channels through sensor reliability estimation, performs asynchronous soft alignment using a learnable temporal kernel, and dynamically balances the contributions of the two modalities through gated fusion. This design reduces the amplification of single-modality perturbations in the fused representation and improves the stability of the prediction function under noise, missing observations, and temporal delays. Consequently, lower regression errors, longer warning lead times, and lower false and missed alarm rates are simultaneously achieved.

### 4.4. Ablation Study

This experiment was designed to verify the practical contributions of semantic modeling, hardware sensor modeling, sensor reliability estimation, asynchronous temporal alignment, bidirectional cross-modal interaction, reliability-aware gated fusion, and multi-task learning by individually removing the key components of HSF-LLMNet.

**Figure 6:**
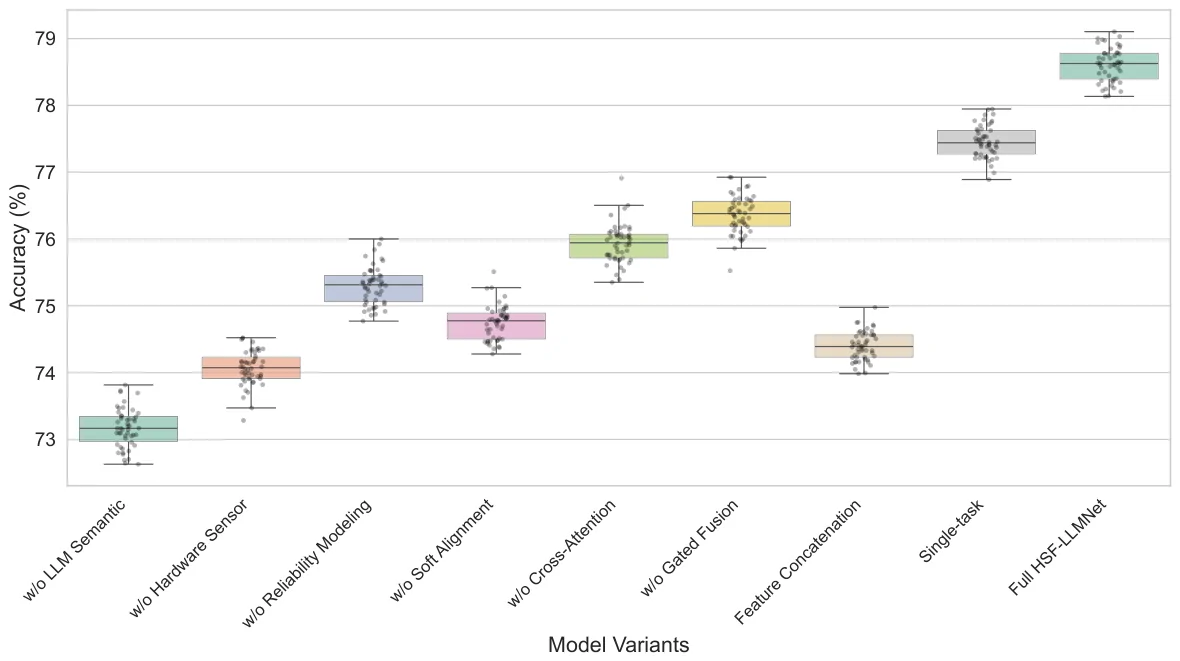
Accuracy distributions of different HSF-LLMNet ablation variants for market direction prediction, with the complete model achieving the highest and most stable performance and confirming the effectiveness of its constituent modules.

As shown in Table 7 and Figure 6, the most pronounced performance degradation was observed when the LLM Semantic Encoder was removed. Accuracy, F1-score, and AUC decreased to 73.24%, 72.51%, and 79.18%, respectively, while RMSE increased to 0.0168. These results indicate that the deep meanings of shocks cannot be effectively identified without event semantics, sentiment intensity, and uncertainty information. When the hardware sensor branch was removed, the Accuracy decreased to 74.06%, confirming that hardware workload, power consumption, and network states provide market pressure information that is independent of textual content. Removing sensor reliability modeling reduced the Accuracy to 75.31% and increased the FAR and MAR to 9.28% and 11.61%, respectively, demonstrating that unweighted abnormal channels and drift noise interfere with prediction. Removing asynchronous soft alignment further degraded the overall performance, confirming that low-frequency textual information and high-frequency sensor signals cannot be directly matched at identical temporal positions. When bidirectional cross-attention and reliable gated fusion were removed, the Accuracy values decreased to 75.89% and 76.37%, respectively, indicating that bidirectional cross-modal interpretation and quality-adaptive weighting provide stable performance gains. Direct feature concatenation achieved an Accuracy of only 74.39%, demonstrating that simple concatenation cannot effectively address temporal misalignment and unequal contributions among heterogeneous modalities.

Single-task training achieved the second-best results, with Accuracy, AUC, and $R^{2}$ values of 77.41%, 83.57%, and 0.795, respectively. However, these results remained lower than the corresponding values of 78.62%, 84.91%, and 0.812 achieved by the complete model, indicating that direction classification, volatility regression, and risk recognition share common market-state information. Full HSF-LLMNet also achieved the lowest RMSE, FAR, and MAR values of 0.0135, 6.82%, and 8.14%, respectively, together with relatively narrow confidence intervals, demonstrating stable performance improvements. From the perspective of the underlying mechanism, the LLM encoder extracts event entities, causal relationships, and risk semantics through high-dimensional attention mappings. Reliability modeling suppresses the influence of abnormal sensors on the joint representation through normalized weights. Asynchronous soft alignment establishes probabilistic associations according to continuous temporal distances and prevents information displacement caused by forced synchronization. Bidirectional cross-attention separately models the asymmetric relationships from events to system responses and from system pressure to event explanations. Gated fusion constrains the multimodal representation within a dynamically weighted feature space and reduces the propagation of low-quality information. Multi-task joint optimization further introduces implicit regularization through shared parameters, enabling the tasks to mutually constrain one another and reducing overfitting. Consequently, the complete model achieves the best overall performance in classification, regression, and risk warning tasks.

### 4.5. Effect of Local Detrending

An ablation experiment was conducted to determine whether direct moving-mean subtraction removed useful slow variations and whether retaining the level, baseline, and residual improved prediction. Four preprocessing variants were evaluated under the same model architecture and training configuration. “No detrending” used only the original minute-level statistics; “moving-mean residual only” reproduced the previous formulation by subtracting a 60 min moving mean and discarding the level term; “level and residual” retained the original level and detrended residual but omitted the estimated baseline and its change; and the proposed method retained the level, causal baseline, residual, and their separate changes. The results are reported in Table 8 as mean ± standard deviation across five time-series folds.

As shown in Table 8, using only the moving-mean residual produced the lowest performance, indicating that direct subtraction discarded slowly varying information relevant to workload and thermal responses. Retaining the original level together with the residual improved all three tasks, while the complete level–baseline–residual representation achieved the best performance. These results support treating local detrending as a feature decomposition operation rather than replacing the original signal with a high-pass residual.

### 4.6. Look-Back Window Sensitivity Analysis

The influence of the textual and hardware look-back lengths was evaluated using the validation portions of the five time-series folds. The candidate textual windows were $L_{d}\in \left\{6,12,24,48\right\}$ h, and the candidate hardware windows were $L_{x}\in \left\{15,30,60,120\right\}$ min. All candidate combinations used the same model architecture, optimization settings, prediction horizon, and early-stopping criterion. Model selection was based on the validation multitask loss, and the test portions were not used to determine $L_{d}$ or $L_{x}$. Table 9 reports the representative validation results as mean ± standard deviation across the five folds.

As shown in Table 9, the combination $L_{d}=24$ h and $L_{x}=60$ min achieved the best overall validation performance and was therefore used in the final experiments. Reducing $L_{d}$ to 6 or 12 h excluded events whose effects continued across several trading hours, whereas extending it to 48 h introduced older events with limited relevance to the current prediction origin. For the hardware modality, windows shorter than 60 min provided insufficient context for workload, power, network, and thermal state evolution. Increasing $L_{x}$ to 120 min introduced less relevant historical operating states and slightly reduced performance. These results also confirm that the two modalities do not require equal look-back lengths: the 24 h textual window captures sparse event history, while the 60 min hardware window characterizes recent infrastructure responses, and their cross-frequency relationship is learned by the asynchronous soft-alignment module.

### 4.7. Computational Efficiency and Deployment Feasibility

The computational evaluation separately considered offline LLM-based semantic extraction and the subsequent training and deployment of the multimodal fusion network. This distinction is necessary because the frozen LLM was not included in the gradient-based optimization illustrated in Figure 3. Instead, the LLM processed each canonical text offline, and its output was stored as a fixed 768-dimensional semantic vector together with the sentiment probabilities, event-category probabilities, risk intensity, and calibrated semantic uncertainty. During fusion-network training, these cached representations were loaded directly, and no activation or gradient was retained for the 7.6-billion-parameter LLM.

The raw record counts in Table 2 do not correspond to the number of independent Transformer inputs. Approximately 2.50 million market records and 38,421,600 native sensor observations were first cleaned, aggregated into one-minute hardware vectors, and aligned using the 60 min hardware look-back window and the 24 h textual look-back window. Prediction origins lacking a valid future target or both usable modalities were removed. This process produced $N_{q}=$ 169,208 aligned 30 min prediction samples. Each sample contained at most 64 cached semantic vectors and 60 one-minute hardware vectors. Therefore, the hardware Transformer and cross-modal attention modules operated on bounded sequences of at most 64×60 event–hardware pairs rather than independently processing every native sensor or market record.

For offline semantic extraction, Qwen2.5-7B-Instruct, containing approximately 7.6 billion parameters, was used as the semantic backbone. Its parameters remained frozen throughout the study. The configured maximum input length was 2048 tokens, and texts exceeding this limit were truncated by retaining the title, the first 1536 tokens, and the final 512 tokens. Offline semantic annotation used K=3 prompt-equivalent inference rounds with greedy decoding, temperature =0, top-p=1.0, and a maximum generated length of 96 tokens. Inference was performed in bfloat16 precision on one NVIDIA GeForce RTX 4090 GPU with 24 GB of memory and a batch size of 8. Operator-level FLOPs were estimated using the PyTorch profiler with the average observed token length. Timing measurements excluded model loading, used 100 warm-up batches, and were repeated five times. The results are reported in Table 10.

As shown in Table 10, freezing the LLM did not imply that no forward inference was performed. The three inference rounds were used only during offline annotation to estimate prompt consistency and semantic uncertainty. The resulting representations were computed once for each canonical text and reused across training epochs, validation runs, and time-series folds. Consequently, the LLM was not executed repeatedly for each of the 169,208 prediction windows. Historical texts required only cache retrieval, while a newly arriving text required one deterministic LLM inference before its representation was added to the cache.

The trainable fusion network consisted of the semantic projection layers, hardware temporal encoder shown in Figure 2, asynchronous bidirectional interaction module shown in Figure 3, reliability-aware gate, and three prediction heads. The hardware branch used three gated causal convolution layers and $N_{t}=2$ temporal Transformer layers with hidden dimension 256 and eight attention heads. The shared cross-modal dimension was $D_{f}=256$, and the bidirectional cross-attention module used four heads. The complete fusion network contained 22.14 million trainable parameters: 3.26 million in semantic projection and semantic heads, 8.71 million in hardware temporal modeling, and 10.17 !million in cross-modal fusion and multitask output heads.

Fusion network training used PyTorch 2.3, CUDA 12.1, bfloat16 automatic mixed precision, AdamW optimization, a mini-batch size of 64, gradient clipping at 1.0, and early stopping with a patience of 10 epochs. Training was conducted on one NVIDIA GeForce RTX 4090 GPU. FLOPs were calculated for one prediction window with $T_{s}=64$, $T_{h}=60$, and batch size 1. GPU latency was measured after 200 warm-up iterations over 2000 timed iterations. Edge device latency was evaluated on an NVIDIA Jetson AGX Orin 64-GB Developer Kit in MAXN mode. The model was exported using ONNX opset 17 and compiled using TensorRT 10.0 with FP16 precision. Jetson power was measured using tegrastats over a continuous 10 min inference run. Table 11 reports the resulting training and deployment measurements.

As shown in Table 11, the frozen LLM accounted for most of the offline computation but did not contribute to the trainable parameter count or peak memory of the fusion network training stage. The trainable network required approximately 11.6 GB of GPU memory and completed one time-series fold in approximately 2.7 h on a single RTX 4090. Its batch-1 inference latency was approximately 4.2 ms on the RTX 4090 and 12.4 ms after TensorRT FP16 conversion on the Jetson AGX Orin. The TensorRT conversion produced changes of less than 0.1 percentage points in the two classification metrics and a volatility–RMSE difference of approximately $10^{-5}$.

The reported Jetson measurements apply to the cached-feature fusion network rather than to Qwen2.5-7B-Instruct. In the practical deployment configuration, historical semantic features are retrieved from the local cache, and newly published texts are processed by a server-side LLM inference service. A new text adds approximately 94.0±5.7 ms of server-side semantic extraction latency for one deterministic inference, after which the resulting 768-dimensional vector is transmitted to the fusion service and cached. Prediction windows without newly arriving texts require only cache retrieval and fusion inference. Thus, the normal online path remains below 15 ms on the Jetson device, excluding data transfer latency, while the less frequent new-text path adds one LLM forward pass rather than the three-round offline voting procedure.

These measurements distinguish the cost of one-time corpus preparation from repeated multimodal prediction. The 2.50 million market records and tens of millions of native sensor observations are reduced to 169,208 bounded prediction windows before Transformer processing, the 7.6B-parameter LLM is excluded from backpropagation, and only the 22.14M-parameter fusion network is optimized during multitask training. This configuration limits training memory, supports real-time prediction from cached semantic features, and permits deployment of the fusion stage on an edge device without requiring the full LLM to operate on that device.

### 4.8. Semantic-Equivalence Validation of Text Augmentation

An independent validation experiment was conducted to determine whether the accepted augmented texts preserved the meaning, sentiment polarity, factual content, and plausibility of their canonical texts. In each of five repeated evaluations, 400 original–augmented pairs were selected through stratified random sampling, including equal numbers of synonym-based paraphrases and event template rewrites and preserving the sentiment class distribution of the training data. Each pair was independently evaluated by three annotators with financial text analysis experience.

The annotators assessed four criteria. Semantic equivalence indicated whether the original and augmented texts described the same event and market implication. Sentiment preservation indicated whether positive, neutral, negative, or panic-related polarity remained unchanged. Factual slot preservation required identical assets, organizations, quantities, dates, units, and event outcomes. Scenario realism indicated whether the augmented wording could plausibly occur in a financial announcement, news report, or investor discussion. A pair was considered valid for a criterion when at least two annotators assigned a positive judgment. Inter-annotator agreement was measured using Fleiss’ κ. Table 12 reports the automatic acceptance rate and human validation results.

As shown in Table 12, more than 95% of the accepted pairs were judged semantically equivalent, and sentiment polarity was preserved in approximately 97% of the pairs. Factual slot preservation exceeded 99%, indicating that the entity, numerical, and temporal constraints prevented the augmentation procedure from altering the underlying event. Event template rewriting showed a slightly lower semantic equivalence and realism rate than synonym-based paraphrasing because it introduced larger syntactic changes. Nevertheless, its validity remained above 93% after automatic filtering. Fleiss’ κ was 0.82±0.03 for semantic equivalence and 0.86±0.02 for sentiment preservation, indicating substantial agreement among the annotators. Candidates rejected by the similarity, entailment, label consistency, factual slot, or uncertainty checks were not included in training. These results indicate that the augmentation procedure primarily produced alternative expressions of existing events rather than new or contradictory market scenarios, thereby maintaining consistency with the preceding deduplication and data-cleaning process.

### 4.9. Statistical Tests of Forecast Superiority

Statistical tests were added to determine whether the performance differences between HSF-LLMNet and the baseline models could be attributed to variation across temporal splits. For market-direction prediction, the F1-score difference was calculated separately for each of the five rolling-origin folds. Given that the folds used partially overlapping training periods, an ordinary paired *t*-test would underestimate the variance of the mean difference. We therefore used the corrected paired test

(132) $$t_{\mathrm{corr}}=\frac{\bar{\delta }}{\sqrt{\left(\frac{1}{K}+\frac{{\bar{n}}_{\mathrm{test}}}{{\bar{n}}_{\mathrm{train}}}\right)s_{\delta }^{2}}},$$

where $\delta_{k}$ is the difference between HSF-LLMNet and a baseline in fold *k*, K=5, $s_{\delta }^{2}$ is the sample variance of the fold-level differences, and ${\bar{n}}_{\mathrm{test}}/{\bar{n}}_{\mathrm{train}}$ is the mean test-to-training sample-size ratio. For volatility forecasting, the Diebold–Mariano test was applied to squared-error loss. To avoid treating strongly overlapping asset–time windows as independent observations, the squared errors were first averaged across all assets and prediction windows within each trading day. For baseline model *b*, the daily loss differential was defined as

(133) $$d_{b,\tau }=\frac{1}{N_{\tau }}\sum_{q\in Q_{\tau }}\left[{\left(y_{q}-{\hat{y}}_{b,q}\right)}^{2}-{\left(y_{q}-{\hat{y}}_{\mathrm{HSF},q}\right)}^{2}\right],$$

where $Q_{\tau }$ denotes the prediction samples on trading day τ. The test statistic was

(134) $$DM_{b}=\frac{{\bar{d}}_{b}}{\sqrt{{\hat{\mathrm{Var}}}_{\mathrm{HAC}}\left({\bar{d}}_{b}\right)}},$$

where the variance was estimated using the Newey–West heteroskedasticity- and autocorrelation-consistent estimator with a prespecified lag of five trading days. Positive values of $t_{\mathrm{corr}}$ and $DM_{b}$ indicate superior performance by HSF-LLMNet. All tests were two-sided, and the resulting *p*-values were adjusted using the Holm–Bonferroni procedure separately for the direction prediction and volatility forecasting comparisons. The significance level was fixed at 0.05 before examining the test results.

As shown in Table 13, HSF-LLMNet achieved higher fold-level F1-scores than all representative baselines. After correction for fold dependence and multiple comparisons, the differences remained significant at the 0.05 level. The smallest F1-score improvement was 2.77 percentage points relative to Informer, for which the adjusted *p*-value was 0.047. The improvement over direct feature concatenation was 3.93 percentage points with an adjusted *p*-value of 0.024, indicating that the gain was not explained solely by the addition of sensor and textual features. The Diebold–Mariano statistics were positive for all volatility baselines, indicating that HSF-LLMNet produced lower daily aggregated squared-error losses. The adjusted *p*-values were below 0.001 in all seven comparisons. The RMSE decreased from 0.0158 for the strongest unimodal sensor baseline and 0.0165 for feature concatenation fusion to 0.0135 for HSF-LLMNet. These results provide statistical evidence of forecast improvement beyond comparisons based only on mean performance and standard deviation. The fold-level tests remain supplementary because only five temporal folds were available; the daily loss-differential test provides the primary inference for volatility forecasting while accounting for temporal dependence through HAC variance estimation.

### 4.10. Independent Human Validation of LLM-Derived Semantic Attributes

An independent human-annotation experiment was conducted to evaluate the sentiment polarity, event category, risk intensity, and semantic uncertainty produced by the LLM semantic branch. A total of 2400 canonical texts were sampled from the final chronological holdout period using stratified sampling over text source, publication month, and predicted semantic class. The sample included financial news, corporate disclosures, policy-related texts, and investor discussions. These texts were not used for semantic-head training, prompt selection, uncertainty calibration, hyperparameter tuning, or downstream model training. Qwen2.5-7B-Instruct generated the semantic outputs using the same configuration as the main experiment, including three prompt-equivalent inference rounds, greedy decoding, temperature =0, and top-p=1.0.

Two annotators with experience in financial-text analysis independently labeled every text according to a predefined annotation guide, and a third senior annotator resolved disagreements. Sentiment polarity comprised positive, neutral, negative, and panic-related classes. Event category comprised macroeconomic or policy events, corporate performance or operations, financing or capital operations, regulatory or legal events, market trading or liquidity events, and other events. Risk intensity was annotated as low, medium, or high according to the expected magnitude and immediacy of the market impact. Semantic uncertainty was initially rated on five levels, {0,0.25,0.50,0.75,1.00}, based on ambiguity in event attribution, sentiment direction, causal interpretation, and contextual completeness. For class-level evaluation, uncertainty scores of 0 and 0.25 were grouped as low uncertainty, 0.50 as medium uncertainty, and 0.75 and 1.00 as high uncertainty. Inter-annotator agreement was calculated before adjudication using Cohen’s κ for nominal attributes and weighted Cohen’s κ for ordinal attributes. The agreement coefficients were 0.84 for sentiment polarity, 0.78 for event category, 0.81 for risk intensity, and 0.76 for semantic uncertainty. The adjudicated labels were used as the reference annotations for the precision, recall, and F1-scores reported in Table 14.

As shown in Table 14, the LLM outputs achieved macro-F1 scores of 83.8% for sentiment polarity, 80.4% for event category, 83.5% for risk intensity, and 78.6% for semantic uncertainty. Negative sentiment and corporate performance events obtained the highest class-level F1-scores, whereas panic-related sentiment, other events, and medium uncertainty were more difficult to identify. Manual inspection showed that most panic-related errors involved strongly negative texts without an explicit indication of immediate market disruption, while medium-uncertainty errors mainly arose from texts containing incomplete causal context or conflicting sentiment cues. For the original continuous uncertainty output, the Spearman correlation with the averaged human uncertainty score was 0.71, and the mean absolute error was 0.086. These results support the use of the LLM-generated semantic attributes in the fusion model while also showing that ambiguous and weakly specified texts require uncertainty-based down-weighting.

### 4.11. Incremental Predictive Value Relative to Market-Microstructure Information

To determine whether infrastructure telemetry provides information beyond contemporaneously observable trading activity, an auxiliary market-microstructure benchmark was constructed for the SSE 50, CSI 300, CSI 500, and CSI 1000 indices. Tick-level transaction and five-level limit-order-book records for the corresponding CFFEX index-futures contracts, IH, IF, IC, and IM, were obtained through the Wind Level-2 database for the common multimodal period from 1 March 2024 to 30 June 2025. At the start of each trading day, the active contract was selected using the highest trading volume on the preceding trading day, thereby avoiding the use of future same-day information. After timestamp synchronization and target-availability screening, the auxiliary sample contained 86,544 index–time windows, including 60,580 training windows, 12,982 validation windows, and 12,982 test windows. Tick records were causally aggregated into right-aligned one-minute intervals, and only records timestamped no later than the forecast origin were retained.

The microstructure feature set contained mid-quote returns, quoted and effective spreads, bid and ask depth at levels 1–5, depth imbalance, order-flow imbalance, buyer- and seller-initiated volume, trade intensity, order-arrival intensity, cancellation intensity, microprice deviation, and realized volatility calculated from 5-s mid-quote returns. Each model used a 60-min input window and the same 30-min index-level targets, rolling-origin folds, purge and embargo rules, optimization budget, and validation-based model-selection procedure. The hidden dimension was fixed at 256, and the trainable parameter counts of the neural baselines were restricted to 21.8–22.4 million to prevent model size from determining the comparison. Sensor-inclusive models were tested against their matched no-sensor specifications using the corrected paired fold-level test for directional F1-score and the Diebold–Mariano test with five-day Newey–West variance estimation for volatility forecasts. The resulting *p*-values were adjusted using the Holm–Bonferroni procedure.

As shown in Table 15, replacing the basic price–volume input with tick-derived microstructure variables increased the F1-score from 71.98% to 74.43% and reduced the volatility RMSE from 0.0169 to 0.0152, confirming that order-book and order-flow variables form a stronger benchmark than aggregated market variables alone. Adding textual information further increased the F1-score to 76.84%. Relative to this public information benchmark, direct sensor concatenation produced an F1-score gain of 0.99 percentage points, whereas HSF-LLMNet produced a gain of 2.28 percentage points and reduced RMSE by 0.0017. Both improvements remained significant after correction for multiple comparisons. The residualized sensor specification retained an F1-score improvement of 1.67 percentage points and an RMSE reduction of 0.0013 after the sensor channels were residualized against mid-quote returns, quoted and effective spreads, bid and ask depth, depth imbalance, order-flow imbalance, trade intensity, cancellation intensity, and realized volatility. These results indicate that part of the raw sensor signal reflects contemporaneous trading intensity, but the remaining operational-state variation provides incremental forecasting information beyond the evaluated market-microstructure variables. Since the auxiliary experiment covers four index-futures markets rather than the complete eight-index sample, the conclusion is limited to the instruments and period included in this robustness test.

### 4.12. Operational Risk-Management Evaluation

An operational policy simulation was conducted to evaluate whether the risk warning outputs could reduce infrastructure congestion. The no-intervention baseline retained the original resource configuration throughout each test period. The reactive policy allocated additional resources only after CPU utilization exceeded 85% or end-to-end processing latency exceeded 12 ms. The predictive policy used the HSF-LLMNet high-risk probability and activated resource pre-allocation when the validation-selected warning threshold was exceeded. The intervention increased the available computing quota by 20% and the network bandwidth allocation by 25% for the subsequent 30 min. All thresholds and intervention parameters were fixed using the validation set.

As shown in Table 16, the predictive policy reduced the average infrastructure overload duration from 38.6 to 12.4 min per trading day and reduced the 95th-percentile processing latency from 18.4 to 11.6 ms. Compared with the reactive policy, HSF-LLMNet reduced missed congestion episodes from 18.6% to 8.9%, at the cost of a 1.4-percentage-point increase in energy consumption. These results indicate that the warning output has operational value for advance resource allocation, although they do not establish the profitability of a trading strategy.

### 4.13. Stability Across Market and Infrastructure Regimes

A stratified out-of-sample analysis was conducted to determine whether the incremental value of infrastructure telemetry was stable across assets, market regimes, volatility and trading-volume conditions, intraday periods, major-announcement windows, and infrastructure operating states. The comparison used the pooled predictions from the five rolling-origin test folds, containing 25,381 index–time windows. HSF-LLMNet was compared with a parameter-matched Market+Text model that retained the same market encoder, semantic representations, prediction heads, training folds, forecast origins, and 30 min targets but excluded the hardware branch and cross-modal interaction modules. The Market+Text model contained 22.08 million trainable parameters, compared with 22.14 million for HSF-LLMNet.

All subgroup variables were constructed using information available no later than the prediction origin. For index *a* at origin $T_{q}$, the market regime was determined from the trailing 20-trading-day cumulative return. Bear, sideways, and bull regimes corresponded to values below the 33rd percentile, between the 33rd and 67th percentiles, and above the 67th percentile, respectively. These percentile thresholds were estimated separately from the training portion of each temporal fold. Volatility and trading volume regimes were defined using the trailing 60 min realized volatility and trading volume. Low and high regimes corresponded to observations below the training set 25th percentile and above the training set 75th percentile, respectively, while the remaining observations formed the medium regime.

The opening period covered 09:30–10:00, the middle period covered 10:00–11:30 and 13:00–14:30, and the closing period covered 14:30–15:00. A major-announcement window included prediction origins occurring within 60 min after a macroeconomic-policy announcement, regulatory decision, earnings release, or corporate disclosure. Infrastructure congestion was defined as CPU utilization above 85%, end-to-end processing latency above 12 ms, or packet loss above 1.0% during the 60-min sensor window. Elevated-load conditions included CPU utilization of 70–85%, latency of 8–12 ms, or packet loss of 0.5–1.0%, provided that the congestion criterion was not satisfied. All remaining observations were assigned to the normal infrastructure condition. Table 17 reports the subgroup results calculated from pooled out-of-sample predictions.

As shown in Table 17, HSF-LLMNet improved both directional F1-score and volatility RMSE for all eight indices. The asset-level F1-score improvement ranged from 2.54 percentage points for the SSE 50 to 3.27 percentage points for ChiNext. Larger improvements were observed for STAR 50, CSI 1000, and ChiNext, while the gains remained positive for the broad-market and large-cap indices. The results were therefore not determined by a single index. The sensor contribution varied across market regimes. The F1-score gain increased from 1.65 percentage points in the low-volatility regime to 4.46 percentage points in the high-volatility regime. Similarly, the improvement increased from 1.71 percentage points in the lowest-volume regime to 4.34 percentage points in the highest-volume regime. Within 60 min after a major announcement, adding infrastructure telemetry improved F1 by 4.85 percentage points and reduced RMSE by 0.0037, compared with improvements of 2.15 percentage points and 0.0011 outside announcement windows. The gains were also larger during the opening and closing periods than during the middle of the trading session. The largest differences occurred across infrastructure conditions. Under normal operating conditions, the hardware branch increased F1 by 1.64 percentage points and reduced RMSE by 0.0009. During elevated-load periods, these improvements increased to 3.36 percentage points and 0.0021, respectively. During congestion episodes, the F1-score gain reached 5.11 percentage points and the RMSE reduction reached 0.0044. These results indicate that infrastructure telemetry does not provide a constant advantage across all observations. Its incremental contribution is concentrated in high-volatility, high-volume, announcement-related, market-opening or market-closing, and infrastructure-congestion conditions, when trading demand and platform pressure increase simultaneously.

### 4.14. Separating Market-Induced Infrastructure Pressure from Platform-Specific Noise

Additional control experiments were conducted to determine whether the predictive contribution of the sensor branch originated from market-conditioned infrastructure pressure or from platform-specific operational disturbances. System, container, network, and maintenance logs from the three Dell PowerEdge R750 servers, the NVIDIA Jetson AGX Xavier edge node, and the two Cisco Catalyst C9300-24T switches were synchronized with the sensor timestamps. Operational-noise episodes included scheduled software updates, Docker container restarts, log rotation, disk backup, background CPU or GPU jobs, network diagnostics, sensor calibration, and device maintenance. A prediction window was marked as operationally contaminated when one of these events occurred during its 60 min sensor look-back interval or within the following 10 min recovery period. Using mutually exclusive event labels assigned according to the priority order of maintenance, software restart, network disturbance, storage activity, and background computation, 3482 of the 25,381 pooled test windows were identified as operational-noise windows, while the remaining 21,899 windows were classified as clean.

To remove the directly observable component of platform-specific noise without removing market-conditioned workload variation, each sensor channel was adjusted using a regression fitted only on the training portion of the corresponding temporal fold:

(135) $$x_{t,m}=f_{m}\;\left(z_{t}^{\mathrm{mkt}}\right)+\gamma_{m}^{⊤}o_{t}+\varepsilon_{t,m},\;x_{t,m}^{\mathrm{adj}}=x_{t,m}-{\hat{\gamma }}_{m}^{⊤}o_{t},$$

where $z_{t}^{\mathrm{mkt}}$ contains contemporaneously observable return, trading volume, trading value, realized volatility, liquidity, and text arrival variables, while $o_{t}$ contains platform-only controls, including software update, container restart, backup, background process, network maintenance, sensor calibration, and hardware fault indicators. The adjusted signal $x_{t,m}^{\mathrm{adj}}$ removes the variation explained by recorded operational disturbances while retaining the component associated with market-conditioned workload and unrecorded residual variation. As a negative control, complete 60 min sensor sequences were permuted across trading days within the same index and 30 min intraday time block, preserving their marginal distribution and intraday seasonality while breaking their alignment with the corresponding market window. A matched replay experiment was also performed on 1200 test windows. Each historical workload was executed once under the normal configuration and once with independent nuisance activity consisting of a 16-thread CPU and 8 GB memory load generated by stress-ng, a sequential storage workload generated by fio, and  2.5 Gbit/s background network traffic generated by iperf3. For the network disturbance condition, tc netem additionally introduced 10 ms delay and 1.0% packet loss. Market inputs, request timestamps, labels, and prediction origins were identical across the paired runs. Table 18 reports the resulting comparisons.

As shown in Table 18, excluding windows affected by recorded maintenance, software, storage, network, or background computation events did not remove the sensor contribution. On the 21,899 clean windows, HSF-LLMNet improved F1 by 2.69 percentage points and reduced RMSE by 0.0014 relative to the matched Market+Text model. By contrast, the improvement on the 3482 operational noise windows was limited to 0.51 percentage points in F1 and 0.0004 in RMSE and was not statistically significant. This difference indicates that uncontrolled platform activity can obscure the market-related component of the sensor sequences. After subtracting the component associated with logged operational disturbances, the complete test sample retained an F1 improvement of 2.32 percentage points and an RMSE reduction of 0.0012. The negative-control experiment produced an F1 improvement of only 0.17 percentage points after sensor sequences were permuted across trading days, and the difference was not significant. Therefore, the model did not obtain the reported gain solely from intraday sensor seasonality or persistent device-specific distributions. In the matched replay experiment, injecting background computation, storage traffic, and network interference reduced the raw sensor F1 gain from 2.77 to 0.64 percentage points. Applying the nuisance adjustment recovered an improvement of 2.24 percentage points and an RMSE reduction of 0.0012. These controls do not establish that every remaining sensor fluctuation is caused by market activity, because unrecorded operational events may still exist. They show, however, that the main predictive gain is not explained entirely by logged maintenance activity, background workloads, device-specific distributions, or experimentally injected platform noise.

### 4.15. Matched Information Set Comparison

To explicitly evaluate the incremental contribution of different information sources, we conducted a matched information set comparison using five configurations: Market-only, Market+Text, Market+Sensor, Market+Text+Sensor with direct feature concatenation, and the complete HSF-LLMNet. All configurations used the same prediction origins, chronological data partitions, rolling-origin evaluation protocol, 30 min prediction targets, and preprocessing procedures. The Market-only configuration used only historical market variables; Market+Text additionally incorporated LLM-derived semantic information; Market+Sensor incorporated infrastructure telemetry instead of text; Market+Text+Sensor (Direct Concatenation) used all three information sources but combined them through direct feature concatenation; and HSF-LLMNet used the same three information sources together with the proposed asynchronous alignment, reliability modeling, and gated fusion mechanism. This comparison was designed to distinguish the incremental value of textual and sensor information from the additional contribution of the proposed fusion architecture.

The results in Table 19 allow the incremental contribution of each information source to be assessed directly. In particular, Market-only versus Market+Text evaluates the contribution of semantic information, Market-only versus Market+Sensor evaluates the additional value of infrastructure telemetry, and Market+Text+Sensor (Direct Concatenation) versus HSF-LLMNet evaluates whether the proposed asynchronous reliability-aware fusion strategy provides benefits beyond simply combining the same input modalities.

### 4.16. Discussion

The practical interpretation of HSF-LLMNet depends on the information set available to its user. The textual and market branches rely on public announcements, news, investor discussions, prices, returns, trading volume, trading value, realized volatility, and liquidity variables. In contrast, the hardware branch uses platform-side operational telemetry, including request processing workload, CPU and GPU utilization, memory activity, network latency, packet loss, power consumption, and temperature. These variables are generally unavailable to public investors and should not be treated as publicly observable market predictors. Accordingly, the present study positions HSF-LLMNet primarily as a framework for internal market-state sensing and infrastructure-aware risk management, rather than as a general market-forecasting system that can be directly reproduced by external investors.

The intended users are exchanges, securities brokers, market data providers, institutional trading platforms, and financial infrastructure operators that collect telemetry from their own computing and communication systems. Within these organizations, the model can support short-horizon market risk monitoring, operational risk assessment, computational resource allocation, network capacity management, congestion control, request throttling, and supervision of order processing or market data services. For example, a simultaneous increase in predicted market risk, request load, and network latency may trigger additional computing-resource allocation or stricter traffic-control rules. A public investor cannot obtain the complete input set by observing an exchange externally. A market participant could use the framework only when it operates its own order-management, execution, or market-data infrastructure and replaces the experimental sensor channels with telemetry generated by that infrastructure. The manuscript therefore does not claim that external investors can access exchange-internal workload, matching-engine status, or network measurements through public information sources. The predictive contribution of the sensor branch must also be separated from the advantage created by access to operator-owned information. Market-only and market-plus-text models represent the public-information benchmark, whereas market-plus-sensor and complete-fusion models represent the operator-information setting. To determine whether the sensor variables contain information beyond conventional public predictors, the revised experiments control for contemporaneous prices, returns, trading volume, trading value, realized volatility, and liquidity variables. Sensor features are additionally residualized against these market predictors using parameters estimated only from the training portion of each temporal fold, and the final 5 and 10 min of sensor observations are removed in separate robustness tests. Predictive gains that remain after these controls are interpreted as incremental information contained in the platform’s operational response. Gains that disappear are interpreted as evidence that the sensor variables primarily transform or proxy contemporaneous market activity. Even when incremental gains remain, they represent an operator-specific informational advantage rather than an improvement available to all market participants. Due to the fact that the current telemetry was collected from a controlled simulation platform rather than a production exchange, validation using independently operated exchange, broker, or institutional platform data is required before the findings can be generalized to real trading infrastructures.

### 4.17. Limitations and Future Work

Several limitations remain in the present study. First, the hardware sensor data were primarily collected from a simulated trading-infrastructure platform. Differences may therefore exist between the experimental environment and production stock exchanges, brokerage data centers, market-data platforms, and institutional trading systems in terms of server configurations, network topologies, workload distributions, and request-processing patterns. Consequently, the generalization capability of HSF-LLMNet across independently operated infrastructures requires further validation. Second, the empirical task should be interpreted as minute-resolution short-horizon intraday forecasting rather than high-frequency trading in the conventional market-microstructure sense. Market observations were sampled at one-minute intervals, native hardware measurements were aggregated into right-aligned one-minute intervals, and the principal prediction horizon was 30 min, with 60 min considered only in the horizon-sensitivity analysis. The dataset does not contain tick-by-tick transactions, message-level order arrivals and cancellations, trade direction, queue position, bid–ask spreads, market depth, or complete limit-order-book snapshots. Therefore, the reported results do not establish applicability to sub-second prediction, ultra-low-latency execution, or order-level trading decisions.

Third, the relationships among textual events, market conditions, and hardware operating states should not be interpreted as direct causal relationships. Trading pressure may affect request load and infrastructure utilization, but equipment maintenance, network switching, software updates, background processes, and hardware faults may generate similar sensor variations. The reliability-aware module reduces the influence of missing, drifting, or isolated abnormal measurements, but it cannot establish that all retained sensor variation originates from market activity. In addition, event categories, risk intensity, and semantic uncertainty generated by the large language model may remain sensitive to prompt design, financial terminology, contextual completeness, and information-dissemination bias. The present findings should therefore be interpreted as evidence of predictive association within a controlled minute-level environment rather than as evidence of a structural causal relationship.

Future work will evaluate the framework using synchronized operational data from real data centers, edge market-data nodes, broker systems, and trading platform infrastructures. To extend the analysis to a market microstructure setting, future datasets will incorporate tick-level transaction prices and timestamps, order arrivals, cancellations, trade direction, bid–ask spreads, multi-level market depth, order-flow imbalance, queue dynamics, and execution latency. These variables will enable comparisons against limit-order-book and order-flow forecasting models and determine whether infrastructure telemetry provides incremental information beyond a complete microstructure information set. Cross-platform experiments will also be conducted across different markets, asset classes, volatility regimes, and infrastructure conditions. Causal inference, anomaly tracing, and domain-adaptation methods will be investigated to separate market-induced computational pressure from maintenance operations, hardware failures, and unrelated workloads. Lightweight deployment, online updating, and model-interpretation mechanisms will additionally be developed to support continuous intraday risk monitoring, resource scheduling, congestion control, and emergency response in practical financial infrastructures.

## 5. Conclusions

With the rapid development of intelligent trading platforms, data centers, and edge computing nodes, market risk is no longer manifested solely through price fluctuations and textual sentiment changes but is also reflected in infrastructure operating states, including server workload, device power consumption, network latency, and packet loss rate. HSF-LLMNet is proposed to reformulate short-term market volatility prediction as an intelligent sensing task jointly driven by semantics and hardware sensor signals. Large language models are employed to extract event categories, sentiment polarity, risk intensity, and semantic uncertainty. Reliable temporal modeling is introduced to suppress sensor missingness, drift, and abnormal noise, while asynchronous soft alignment, bidirectional cross-attention, and reliability-aware gated fusion are used to address temporal misalignment and quality discrepancies between low-frequency irregular texts and high-frequency continuous hardware signals. The experimental results demonstrate that HSF-LLMNet achieved an Accuracy of 78.62%, a Precision of 78.14%, a Recall of 77.83%, an F1-score of 77.98%, an AUC of 84.91%, and an MCC of 57.36% for market direction prediction over the subsequent 30 min, consistently outperforming linear models, deep temporal models, unimodal approaches, and conventional feature-concatenation methods. In the volatility prediction and risk warning tasks, the MAE, RMSE, and MAPE were reduced to 0.0089, 0.0135, and 9.21%, respectively, while $R^{2}$ increased to 0.812. The average effective warning time reached 15.37 min, and the false alarm rate and missed alarm rate were reduced to 6.82% and 8.14%, respectively. The ablation results further verified the effectiveness of semantic encoding, sensor reliability modeling, asynchronous alignment, bidirectional interaction, gated fusion, and multi-task learning. These findings indicate that semantic shocks and infrastructure responses provide strongly complementary information and can offer reliable support for market state perception, resource scheduling, and risk warning in intelligent trading systems, data centers, and edge market data nodes.

## Figures and Tables

**Table 1:** Comparison of HSF-LLMNet with closely related financial forecasting approaches.

Study or Approach	Prediction Target	Inputs	Alignment/Reliability Modeling	Multi-Task
LLM-based market sentiment prediction [19]	Market sentiment	Financial news, LLM-derived sentiment, and market variables	Feature-level combination; no sensor reliability or explicit cross-frequency alignment	No
Text-driven financial forecasting [22,25,26]	Market behavior or volatility	Financial texts and conventional market variables	Fixed-window aggregation or representation-level fusion	Generally no
RiskLabs [40]	Volatility and variance	Earnings-call text, news, vocal information, and market time series	Event-centered alignment; no infrastructure-sensor reliability modeling	Yes
General multimodal market forecasting [38,39]	Market prediction or financial risk	Financial text, market time series, and macroeconomic variables	Attention- or representation-based multimodal fusion	Method-dependent
HSF-LLMNet	30-min direction, volatility, and risk	Financial text, market variables, and infrastructure telemetry	Learnable asynchronous alignment, sensor-reliability modeling, and semantic-uncertainty-aware fusion	Yes

**Table 2:** Dataset composition and usable prediction windows. Raw and cleaned counts refer to the complete source archives, whereas aligned window counts refer only to the common multimodal interval from 1 March 2024 to 30 June 2025. Modality-specific aligned counts may overlap.

Data Type	Sampling	Source or Acquisition Configuration	Raw Records	Cleaned Records	Aligned Windows
News and corporate announcements	Event-driven	Sina Finance, Eastmoney, CNINFO, and CSRC disclosure platform	128,436	119,842	87,436
Investor discussion texts	Event-driven	Five public investor communities	93,517	84,963	93,118
Index market data	1 min	Eight study indices; price, return, volume, value, volatility, and liquidity variables	2,517,840	2,431,776	171,024
Computational resource state	1 Hz	Three Dell PowerEdge R750 nodes and one NVIDIA Jetson AGX Xavier; 24 channels	4,260,000	4,071,382	168,944
Power and electrical state	2 Hz	Three INA219 and three INA226 monitoring modules; 12 channels	5,034,816	4,836,205	168,736
Environmental conditions	0.2 Hz	Six Sensirion SHT35 temperature/humidity sensors; 12 channels	1,214,352	1,189,744	169,008
Network communication state	1 Hz	Two Cisco C9300-24T switches and two network probes; 16 channels	8,126,400	7,692,118	168,520
Equipment vibration state	10 Hz	Four MPU6050 inertial sensors; 12 channels	1,536,000	1,472,603	168,112
All hardware sensor modalities	0.2–10 Hz	24 devices or modules; 76 channels	20,171,568	19,262,052	169,208
Final aligned multimodal dataset	30 min origins	Eight index-level prediction targets	–	–	169,208

**Table 3:** Agreement and calibration results for the semantic uncertainty estimator. The numerical values are reported as mean ± standard deviation across five folds.

Metric	Result
Weighted Cohen’s κ between human annotators	0.78±0.03
Spearman correlation between $u_{i}$ and human scores	0.73±0.02
Mean absolute error relative to human scores	0.081±0.006
Brier score	0.067±0.005
Expected calibration error before calibration	0.074±0.006
Expected calibration error after calibration	0.028±0.004

**Table 4:** Inputs to the reliability-aware gating network.

Signal	Dimension	Definition
${\bar{r}}_{q}^{s}$	$D_{f}$	Window-level semantic interaction representation from Equation (97).
${\bar{r}}_{q}^{h}$	$D_{f}$	Window-level hardware interaction representation from Equation (98).
${\bar{u}}_{q}$	1	Attention-weighted mean of the event-level semantic uncertainty scores.
${\bar{\rho }}_{q}$	1	Coverage-weighted mean of the absolute channel-reliability scores.
$n_{q}$	1	Text density normalized by the maximum retained number of events.
$c_{q}$	1	Cross-modal consistency obtained from pooled-vector similarity and bidirectional alignment agreement.
$m_{q}^{s},m_{q}^{h}$	1 each	Binary indicators of semantic and hardware modality availability.

**Table 5:** Performance comparison of different methods for predicting market direction over the subsequent 30 min. The results are reported as the mean ± standard deviation.

Method	Accuracy (%) ↑	Precision (%) ↑	Recall (%) ↑	F1-Score (%) ↑	AUC (%) ↑	MCC (%) ↑
Logistic Regression	61.28±0.92	60.91±1.01	59.84±1.13	60.37±0.98	65.42±0.88	22.36±1.42
XGBoost	66.35±0.76	65.92±0.83	65.14±0.97	65.53±0.79	71.26±0.73	32.94±1.19
LSTM	68.45±0.78	67.91±0.84	67.36±0.88	67.63±0.81	73.84±0.76	37.08±1.17
Transformer	70.86±0.63	70.28±0.69	69.91±0.77	70.09±0.67	76.49±0.62	41.92±0.97
Informer	71.34±0.60	70.91±0.65	70.36±0.73	70.63±0.62	77.08±0.58	42.88±0.92
Text-only FinBERT	70.13±0.71	69.68±0.76	69.17±0.84	69.42±0.73	75.92±0.68	40.51±1.07
LLM-text-only	72.46±0.62	72.08±0.67	71.42±0.76	71.75±0.65	78.31±0.60	45.16±0.96
Sensor-only Transformer	69.28±0.77	68.74±0.83	68.11±0.91	68.42±0.81	74.83±0.74	38.76±1.15
Feature Concatenation Fusion	$\underline{74.39\pm 0.55}$	$\underline{73.91\pm 0.58}$	$\underline{73.46\pm 0.65}$	$\underline{73.68\pm 0.56}$	$\underline{80.34\pm 0.52}$	$\underline{48.91\pm 0.83}$
HSF-LLMNet	78.62±0.46	78.14±0.50	77.83±0.56	77.98±0.47	84.91±0.43	57.36±0.72

**Table 6:** Performance comparison of different methods for volatility prediction and risk warning over the subsequent 30 min. The results are reported as the mean ± standard deviation.

Method	MAE ↓	RMSE ↓	MAPE (%) ↓	$R^{2}$↑	EWT (min) ↑	FAR (%) ↓	MAR (%) ↓
Logistic/Linear Regression	0.0187±0.0010	0.0264±0.0014	19.82±0.88	0.412±0.026	6.42±0.46	18.73±1.04	22.46±1.13
XGBoost	0.0152±0.0007	0.0218±0.0010	15.94±0.73	0.548±0.021	8.36±0.38	14.38±0.86	17.92±0.94
LSTM	0.0139±0.0006	0.0202±0.0009	14.48±0.65	0.598±0.019	9.27±0.35	13.14±0.78	16.37±0.84
Transformer	0.0126±0.0005	0.0184±0.0007	12.97±0.55	0.657±0.016	10.34±0.31	11.63±0.66	14.47±0.73
Informer	0.0123±0.0005	0.0180±0.0007	12.61±0.52	0.671±0.015	10.68±0.29	11.29±0.63	14.03±0.71
Text-only FinBERT	0.0134±0.0006	0.0195±0.0009	13.87±0.60	0.619±0.017	9.91±0.34	12.54±0.72	15.68±0.81
LLM-text-only	0.0121±0.0005	0.0177±0.0007	12.38±0.51	0.683±0.015	11.26±0.27	10.93±0.61	13.46±0.67
Sensor-only Transformer	0.0130±0.0006	0.0189±0.0007	13.42±0.57	0.641±0.016	10.47±0.31	11.84±0.67	14.72±0.74
Feature Concatenation Fusion	$\underline{0.0108\pm 0.0004}$	$\underline{0.0161\pm 0.0006}$	$\underline{11.06\pm 0.43}$	$\underline{0.738\pm 0.012}$	$\underline{12.48\pm 0.25}$	$\underline{9.46\pm 0.52}$	$\underline{11.87\pm 0.58}$
HSF-LLMNet	0.0089±0.0004	0.0135±0.0005	9.21±0.38	0.812±0.010	15.37±0.22	6.82±0.43	8.14±0.48

**Table 7:** Ablation results for different components of HSF-LLMNet. The results are reported as the mean ± standard deviation.

Model Variant	Accuracy (%) ↑	F1-Score (%) ↑	AUC (%) ↑	MCC (%) ↑	RMSE ↓	$R^{2}$↑	FAR (%) ↓	MAR (%) ↓
w/o LLM Semantic Encoder	73.24±0.61	72.51±0.63	79.18±0.57	46.38±0.91	0.0168±0.0006	0.724±0.014	10.73±0.57	13.29±0.66
w/o Hardware Sensor Branch	74.06±0.58	73.32±0.61	80.04±0.55	47.96±0.87	0.0161±0.0006	0.741±0.012	9.91±0.55	12.47±0.62
w/o Sensor Reliability Modeling	75.31±0.55	74.68±0.57	81.27±0.52	50.42±0.83	0.0154±0.0006	0.759±0.012	9.28±0.52	11.61±0.60
w/o Asynchronous Soft Alignment	74.72±0.57	74.01±0.60	80.58±0.53	49.18±0.86	0.0158±0.0006	0.748±0.012	9.74±0.53	12.06±0.61
w/o Bidirectional Cross-Attention	75.89±0.52	75.27±0.55	81.93±0.50	51.61±0.79	0.0150±0.0005	0.771±0.011	8.83±0.50	10.92±0.57
w/o Reliable Gated Fusion	76.37±0.51	75.81±0.53	82.46±0.48	52.58±0.77	0.0147±0.0005	0.779±0.011	8.46±0.48	10.41±0.55
Feature Concatenation	74.39±0.55	73.68±0.56	80.34±0.52	48.91±0.83	0.0161±0.0006	0.738±0.012	9.46±0.52	11.87±0.58
Single-task Training	$\underline{77.41\pm 0.48}$	$\underline{76.79\pm 0.51}$	$\underline{83.57\pm 0.46}$	$\underline{54.93\pm 0.74}$	$\underline{0.0141\pm 0.0005}$	$\underline{0.795\pm 0.011}$	$\underline{7.61\pm 0.46}$	$\underline{9.23\pm 0.52}$
Full HSF-LLMNet	78.62±0.46	77.98±0.47	84.91±0.43	57.36±0.72	0.0135±0.0005	0.812±0.010	6.82±0.43	8.14±0.48

**Table 8:** Ablation results for sensor detrending and feature decomposition.

Sensor Preprocessing Method	Direction F1 (%)	Volatility RMSE	Risk AUC (%)
No detrending	77.31±0.52	0.0139±0.0003	84.25±0.44
Moving-mean residual only	76.94±0.61	0.0141±0.0004	83.88±0.51
Original level and residual	77.64±0.47	0.0137±0.0003	84.56±0.41
Level, baseline, and residual	77.98±0.44	0.0135±0.0002	84.91±0.39

**Table 9:** Sensitivity of HSF-LLMNet to textual and hardware look-back window lengths. Results are reported on the validation portions as mean ± standard deviation.

$L_{d}$	$L_{x}$	Direction F1 (%)	Volatility RMSE	Risk AUC (%)
6 h	30 min	75.84±0.68	0.0146±0.0004	82.31±0.57
12 h	60 min	77.12±0.55	0.0139±0.0003	83.75±0.46
24 h	30 min	77.36±0.48	0.0137±0.0003	84.02±0.43
24 h	60 min	77.98±0.44	0.0135±0.0002	84.91±0.39
24 h	120 min	77.51±0.50	0.0138±0.0003	84.38±0.45
48 h	60 min	77.21±0.61	0.0140±0.0004	83.96±0.52

**Table 10:** Configuration and computational cost of offline LLM-based semantic extraction. Timing, memory, and throughput results are reported as mean ± standard deviation.

Item	Configuration or Measured Result
LLM backbone	Qwen2.5-7B-Instruct, approximately 7.6B frozen parameters
Trainable LLM parameters	0
Numerical precision	bfloat16
Configured maximum input length	2048 tokens
Average input length	286.4±137.2 tokens per text
Offline inference rounds	K=3 prompt-equivalent forward passes
Decoding strategy	Greedy decoding, temperature =0, top-p=1.0
Maximum generated length	96 tokens
Inference hardware	NVIDIA GeForce RTX 4090, 24-GB GPU memory
Batch size	8 texts
Number of cleaned canonical texts	204,805
Total number of LLM forward passes	614,415
Estimated computation per text per round	5.92±2.08 TFLOPs
Average latency per text per round	94.0±5.7 ms
Average three-round latency per text	282.0±17.1 ms
Effective inference throughput	10.64±0.61 texts/s
Peak allocated GPU memory	19.18±0.21 GB
Total offline GPU time	16.04±0.92 GPU hours
Cached semantic representation	768-dimensional float16 vector and structured semantic attributes
Cache format and indexing	LMDB indexed by SHA-256 text hash and event ID
Total semantic cache size	0.46±0.01 GB
Average cached-feature retrieval time	0.31±0.04 ms per text

**Table 11:** Computational efficiency of the trainable fusion network and its deployment configurations. Latency is measured with batch size 1 and excludes offline LLM feature extraction and network communication.

Item	Configuration or Measured Result
Aligned prediction samples	169,208 windows
Maximum semantic sequence length	64 cached event vectors
Hardware sequence length	60 one-minute vectors
Trainable fusion network parameters	22.14 M
Semantic projection parameters	3.26 M
Hardware temporal modeling parameters	8.71 M
Cross-modal fusion and output parameters	10.17 M
Computation per prediction window	4.86 GFLOPs
Training hardware	NVIDIA GeForce RTX 4090, 24-GB GPU memory
Training numerical precision	bfloat16 automatic mixed precision
Training batch size	64 windows
Peak training GPU memory	11.62±0.18 GB
Average epochs before early stopping	57.4±6.1
Training time per time-series fold	2.73±0.16 h
Total training time for five folds	13.65±0.81 GPU hours
RTX 4090 PyTorch latency	4.18±0.21 ms/window
RTX 4090 PyTorch throughput	239.2±11.8 windows/s
Jetson ONNX Runtime FP16 latency	20.83±0.74 ms/window
Jetson TensorRT FP16 latency	12.36±0.43 ms/window
Jetson TensorRT FP16 throughput	80.91±2.76 windows/s
Jetson TensorRT peak memory	3.28±0.07 GB
Jetson average module power	27.6±1.2 W
TensorRT direction-F1 change	−0.07 percentage points relative to PyTorch
TensorRT risk-AUC change	−0.06 percentage points relative to PyTorch
TensorRT volatility-RMSE change	$+1.0\times 10^{-5}$ relative to PyTorch

**Table 12:** Semantic equivalence validation of the accepted augmented texts. Results are reported as mean ± standard deviation.

Augmentation Method	Automatic Acceptance	Semantic Equivalence	Sentiment Preservation	Factual Preservation	Scenario Realism
Synonym-based paraphrasing	93.8±0.7	96.1±1.0	97.8±0.8	99.4±0.3	95.5±0.9
Event template rewriting	90.6±0.9	94.2±1.2	96.5±0.9	98.9±0.4	93.6±1.1
Overall	92.2±0.8	95.2±1.1	97.2±0.8	99.2±0.4	94.6±1.0

**Table 13:** Statistical comparison of HSF-LLMNet with representative baseline models. Directional performance is tested using the corrected paired test over five rolling-origin folds. Volatility performance is tested using the Diebold–Mariano test on daily aggregated squared-error differentials. Reported *p*-values are adjusted using the Holm–Bonferroni procedure.

Model	F1-Score (%)	ΔF1 (pp)	$t_{\mathrm{corr}}$	Adjusted *p*	RMSE	DM Statistic	Adjusted *p*
LightGBM	71.42	6.56	7.84	0.009	0.0194	6.72	<0.001
LSTM	73.16	4.82	6.21	0.014	0.0176	5.84	<0.001
Transformer	74.88	3.10	4.18	0.034	0.0167	4.91	<0.001
Informer	75.21	2.77	3.76	0.047	0.0164	4.36	<0.001
LLM-text-only	75.04	2.94	4.02	0.039	0.0161	4.68	<0.001
Sensor-only Transformer	74.17	3.81	5.31	0.021	0.0158	4.27	<0.001
Feature-concatenation fusion	74.05	3.93	5.08	0.024	0.0165	4.73	<0.001
HSF-LLMNet	77.98	—	—	—	0.0135	—	—

**Table 14:** Independent human validation of the semantic attributes generated by the LLM branch. Support denotes the number of manually adjudicated texts in each class. The overall rows report macro-averaged precision, recall, and F1-score across the corresponding classes.

Semantic Attribute	Class	Support	Precision (%)	Recall (%)	F1-Score (%)
Sentiment polarity	Positive	568	85.9	84.5	85.2
	Neutral	736	83.6	86.8	85.2
	Negative	824	88.2	87.0	87.6
	Panic-related	272	80.4	74.6	77.4
	Macro average	2400	84.5	83.2	83.8
Event category	Macroeconomic or policy	412	88.1	85.4	86.7
	Corporate performance or operations	604	87.2	89.0	88.1
	Financing or capital operations	286	79.3	75.2	77.2
	Regulatory or legal	368	84.7	81.8	83.2
	Market trading or liquidity	418	78.1	74.2	76.1
	Other events	312	72.8	69.2	71.0
	Macro average	2400	81.7	79.1	80.4
Risk intensity	Low	868	87.8	90.1	88.9
	Medium	911	79.2	76.8	78.0
	High	621	85.1	82.3	83.7
	Macro average	2400	84.0	83.1	83.5
Semantic uncertainty	Low	1214	86.3	88.7	87.5
	Medium	702	71.8	68.9	70.3
	High	484	80.6	75.8	78.1
	Macro average	2400	79.6	77.8	78.6

**Table 15:** Incremental predictive value of infrastructure telemetry relative to market-microstructure benchmarks. Values are reported as the mean ± standard deviation across five rolling-origin folds. ΔF1 and ΔRMSE are calculated relative to the matched no-sensor model. For Microstructure+Sensor, the reference is Microstructure-only; for the remaining sensor-inclusive models, the reference is Microstructure+Text. The reported *p*-values are Holm–Bonferroni adjusted.

Model and Information Set	Accuracy (%)	F1-Score (%)	AUC (%)	MAE	RMSE	ΔF1 (pp)	ΔRMSE	Adjusted *p* F1/DM
Basic market Transformer	72.43±0.68	71.98±0.71	78.12±0.63	0.0108±0.0003	0.0169±0.0004	–	–	–
Microstructure-only Transformer	75.06±0.59	74.43±0.62	81.02±0.55	0.0099±0.0003	0.0152±0.0003	–	–	–
Microstructure+Text	77.28±0.53	76.84±0.56	83.17±0.49	0.0095±0.0002	0.0146±0.0003	–	–	–
Microstructure+Sensor	77.71±0.51	77.36±0.54	83.54±0.47	0.0093±0.0002	0.0142±0.0003	+2.93	−0.0010	0.018/0.006
Microstructure+Text+Sensor concatenation	78.22±0.49	77.83±0.51	84.06±0.45	0.0091±0.0002	0.0139±0.0003	+0.99	−0.0007	0.041/0.023
HSF-LLMNet with residualized sensors	78.89±0.46	78.51±0.48	84.57±0.42	0.0088±0.0002	0.0133±0.0002	+1.67	−0.0013	0.029/0.011
HSF-LLMNet	79.51±0.43	79.12±0.45	85.26±0.40	0.0085±0.0002	0.0129±0.0002	+2.28	−0.0017	0.013/0.004

**Table 16:** Operational value of HSF-LLMNet risk warnings under a resource-allocation simulation. Reported values are calculated from the pooled out-of-sample test period.

Policy	Overload Duration (min/day)	95th-Percentile Latency (ms)	Packet-Loss Rate (%)	Missed Congestion Episodes (%)	Additional Energy (%)
No intervention	38.6	18.4	1.92	100.0	0.0
Reactive threshold	21.7	14.2	1.21	18.6	4.7
HSF-LLMNet predictive policy	12.4	11.6	0.74	8.9	6.1

**Table 17:** Stability of the incremental sensor contribution across assets, market regimes, trading periods, announcement windows, and infrastructure conditions. The Market+Text model is the matched no-sensor reference. Positive ΔF1 and ΔRMSE values indicate improvements from adding the hardware branch, where $\Delta \mathrm{RMSE}={\mathrm{RMSE}}_{\mathrm{Market}+\mathrm{Text}}-{\mathrm{RMSE}}_{\mathrm{HSF}}$. Results are calculated from pooled out-of-sample predictions.

Stratification Dimension	Subgroup	Test Windows	Market+Text F1 (%)	HSF-LLMNet F1 (%)	ΔF1 (pp)	Market+Text RMSE	HSF-LLMNet RMSE	ΔRMSE
*Asset-level results*								
Asset	SSE Composite	3176	75.12	77.84	2.72	0.0147	0.0134	0.0013
Asset	SSE 50	3194	75.48	78.02	2.54	0.0145	0.0131	0.0014
Asset	CSI 300	3158	75.66	78.31	2.65	0.0143	0.0129	0.0014
Asset	STAR 50	3168	73.89	77.10	3.21	0.0161	0.0142	0.0019
Asset	CSI 500	3162	74.91	77.63	2.72	0.0152	0.0138	0.0014
Asset	CSI 1000	3167	74.32	77.28	2.96	0.0157	0.0140	0.0017
Asset	Shenzhen Component	3174	75.03	77.81	2.78	0.0149	0.0135	0.0014
Asset	ChiNext	3182	74.11	77.38	3.27	0.0159	0.0141	0.0018
*Market and volatility regimes*								
Trailing 20-day return	Bear	8354	74.10	77.48	3.38	0.0160	0.0140	0.0020
Trailing 20-day return	Sideways	8612	75.52	77.67	2.15	0.0142	0.0132	0.0010
Trailing 20-day return	Bull	8415	75.19	78.12	2.93	0.0146	0.0133	0.0013
Trailing 60-min volatility	Low	6345	76.03	77.68	1.65	0.0115	0.0108	0.0007
Trailing 60 min volatility	Medium	12,691	75.23	77.74	2.51	0.0145	0.0133	0.0012
Trailing 60 min volatility	High	6345	72.88	77.34	4.46	0.0198	0.0163	0.0035
Trailing 60 min volume	Low	6346	75.70	77.41	1.71	0.0132	0.0125	0.0007
Trailing 60 min volume	Medium	12,689	75.11	77.69	2.58	0.0148	0.0135	0.0013
Trailing 60 min volume	High	6346	73.12	77.46	4.34	0.0189	0.0157	0.0032
*Trading period and announcement condition*								
Intraday period	Opening	4228	73.58	77.42	3.84	0.0176	0.0148	0.0028
Intraday period	Middle	16,925	75.42	77.71	2.29	0.0141	0.0130	0.0011
Intraday period	Closing	4228	73.91	77.88	3.97	0.0182	0.0150	0.0032
Major announcement	Outside 60 min window	21,519	75.44	77.59	2.15	0.0144	0.0133	0.0011
Major announcement	Within 60 min window	3862	72.86	77.71	4.85	0.0201	0.0164	0.0037
*Infrastructure operating condition*								
Infrastructure condition	Normal	14,570	75.66	77.30	1.64	0.0138	0.0129	0.0009
Infrastructure condition	Elevated load	7211	74.41	77.77	3.36	0.0162	0.0141	0.0021
Infrastructure condition	Congestion	3600	71.98	77.09	5.11	0.0214	0.0170	0.0044

**Table 18:** Control experiments separating market-conditioned infrastructure pressure from platform-specific operational noise. The Market+Text model is the matched no-sensor reference. Positive ΔF1 and ΔRMSE values indicate improvement from adding sensor information, where $\Delta \mathrm{RMSE}={\mathrm{RMSE}}_{\mathrm{Market}+\mathrm{Text}}-{\mathrm{RMSE}}_{\mathrm{HSF}}$. Adjusted *p*-values use the Holm–Bonferroni procedure.

Evaluation Condition	Windows	Market+Text F1 (%)	HSF-LLMNet F1 (%)	ΔF1 (pp)	Market+Text RMSE	HSF-LLMNet RMSE	ΔRMSE	Adjusted *p* F1/DM
All pooled test windows	25,381	75.44	77.98	2.54	0.0148	0.0135	0.0013	0.018/0.006
Clean windows after log-based exclusion	21,899	75.62	78.31	2.69	0.0145	0.0131	0.0014	0.014/0.004
Logged operational noise windows	3482	73.51	74.02	0.51	0.0172	0.0168	0.0004	0.271/0.184
All windows with nuisance-adjusted sensors	25,381	75.44	77.76	2.32	0.0148	0.0136	0.0012	0.022/0.009
Day-block-permuted sensor negative control	25,381	75.44	75.61	0.17	0.0148	0.0147	0.0001	0.612/0.544
Matched replay under normal operation	1200	75.38	78.15	2.77	0.0146	0.0131	0.0015	0.016/0.005
Matched replay with injected nuisance, raw sensors	1200	75.38	76.02	0.64	0.0146	0.0143	0.0003	0.238/0.171
Matched replay with injected nuisance, adjusted sensors	1200	75.38	77.62	2.24	0.0146	0.0134	0.0012	0.031/0.012

**Table 19:** Matched Information set comparison for the 30 min prediction task.

Model/Information Set	Direction F1 (%) ↑	Volatility RMSE ↓	Risk FAR/MAR (%) ↓
Market-only	72.86	0.0173	11.68/14.36
Market+Text	75.44	0.0148	8.94/10.86
Market+Sensor	74.21	0.0157	9.62/11.74
Market+Text+Sensor (Direct Concatenation)	73.68	0.0161	9.46/11.87
HSF-LLMNet	**77.98**	**0.0135**	**6.82/8.14**

## Data Availability

The data presented in this study are available on request from the corresponding author.

## References

[1] Chen H. (2026). Intelligent Agent-Based Market Research: Cloud-Orchestrated Large Language Models as Financial Analysts. Cloud Comput. Data Sci..

[2] Balakrishnan S.K. (2023). Adaptive AI-driven on-demand packet inspection for low-latency market data connectivity between peer exchanges. J. Comput. Anal. Appl..

[3] Aldasoro I., Hördahl P., Zhu S. (2022). Under pressure: Market conditions and stress. BIS Q. Rev..

[4] Song Z., Rahmadya B., Sun R., Takeda S. (2022). An RFID-based wireless vibration and physical-shock sensing system using edge processing. IEEE Sens. J..

[5] Fei T., Mukhopadhyay S.C., Da Costa J.P.J., RoyChaudhuri C., Lan L., Demitri N. (2024). Spatial environment perception and sensing in automated systems: A review. IEEE Sens. J..

[6] Wu S., Liu Y., Zou Z., Weng T.H. (2022). S_I_LSTM: Stock price prediction based on multiple data sources and sentiment analysis. Connect. Sci..

[7] Qian C., Zhang H., Sha L., Zheng Z. (2025). Hsf: Defending against jailbreak attacks with hidden state filtering. Companion Proceedings of the ACM on Web Conference 2025.

[8] Dongrey S. (2022). Study of market indicators used for technical analysis. Int. J. Eng. Manag. Res..

[9] Zhao S., Hong X., Yang J., Zhao Y., Ding G. (2023). Toward label-efficient emotion and sentiment analysis. Proc. IEEE.

[10] Karri N., Muntala P.S.R.P., Jangam S.K. (2022). Forecasting Hardware Failures or Resource Bottlenecks Before They Occur. Int. J. Emerg. Res. Eng. Technol..

[11] Yabe T., Rao P.S.C., Ukkusuri S.V., Cutter S.L. (2022). Toward data-driven, dynamical complex systems approaches to disaster resilience. Proc. Natl. Acad. Sci. USA.

[12] Abdelsamie M., Wang H. (2024). Comparative analysis of LLM-based market prediction and human expertise with sentiment analysis and machine learning integration. Proceedings of the 2024 7th International Conference on Data Science and Information Technology (DSIT).

[13] Bi J. (2022). Stock market prediction based on financial news text mining and investor sentiment recognition. Math. Probl. Eng..

[14] Herrera-Poyatos D., Peláez-González C., Zuheros C., Herrera-Poyatos A., Tejedor V., Herrera F., Montes R. (2025). An overview of model uncertainty and variability in LLM-based sentiment analysis: Challenges, mitigation strategies, and the role of explainability. Front. Artif. Intell..

[15] Omol E., Mburu L., Onyango D. (2024). Anomaly detection in IoT sensor data using machine learning techniques for predictive maintenance in smart grids. Int. J. Sci. Technol. Manag..

[16] Zhang Y., Fu C., Wang X., Zhang Y., Xiong Z., Pan J., Yin J. (2026). AI-Driven Sensing for Cross-Lingual Risk Prediction via Semantic Alignment and Multimodal Temporal Fusion. Appl. Sci..

[17] Kong Y., Hwang Y., Kaiser M., Vryonides C., Oomen R., Zohren S. Fusing Narrative Semantics for Financial Volatility Forecasting. Proceedings of the 6th ACM International Conference on AI in Finance, Singapore.

[18] Islam T., Sheakh M.A., Sadik M.R., Tahosin M.S., Foysal M.M.R., Ferdush J., Begum M. (2024). Lexicon and deep learning-based approaches in sentiment analysis on short texts. J. Comput. Commun..

[19] Qi R. (2025). Financial news sentiment analysis and market sentiment prediction based on large language models. J. Comput. Signal Syst. Res..

[20] Zhai S., Zhang Z. (2023). Read the news, not the books: Forecasting firms’ long-term financial performance via deep text mining. ACM Trans. Manag. Inf. Syst..

[21] Farimani S.A., Jahan M.V., Milani Fard A. (2022). From text representation to financial market prediction: A literature review. Information.

[22] Wu Y., Sun M., Zheng H., Hu J., Liang Y., Lin Z. (2024). Integrative analysis of financial market sentiment using cnn and gru for risk prediction and alert systems. Proceedings of the 2024 International Conference on Electronics and Devices, Computational Science (ICEDCS).

[23] Kumar R., Kumar H., Shalini K. (2025). Cross-Domain Knowledge Transfer using LLMs and Domain-Specific Knowledge Graphs. Proceedings of the 2025 IEEE International Conference on Emerging Technologies and Applications (MPSec ICETA).

[24] Yan C. (2025). LLM-enhanced multi-causal event causality mining in financial texts. J. King Saud Univ. Comput. Inf. Sci..

[25] Yigit Y., Ferrag M.A., Ghanem M.C., Sarker I.H., Maglaras L.A., Chrysoulas C., Moradpoor N., Tihanyi N., Janicke H. (2025). Generative AI and LLMs for critical infrastructure protection: Evaluation benchmarks, agentic AI, challenges, and opportunities. Sensors.

[26] Pei J., Feng C., Wang P., Tabassum H., Shi D. (2025). Latent diffusion model-enabled low-latency semantic communication in the presence of semantic ambiguities and wireless channel noises. IEEE Trans. Wirel. Commun..

[27] Bin Mofidul R., Alam M.M., Rahman M.H., Jang Y.M. (2022). Real-time energy data acquisition, anomaly detection, and monitoring system: Implementation of a secured, robust, and integrated global IIoT infrastructure with edge and cloud AI. Sensors.

[28] Sun M. (2024). The application of embedded hardware system and blockchain in rural financial management cloud platform. Decis. Mak. Appl. Manag. Eng..

[29] Nourildean S.W., Hassib M.D., Mohammed Y. (2022). Internet of things based wireless sensor network: A review. Indones. J. Electr. Eng. Comput. Sci..

[30] Liu W., Haardt M., Greco M.S., Mecklenbräuker C.F., Willett P. (2023). Twenty-five years of sensor array and multichannel signal processing: A review of progress to date and potential research directions. IEEE Signal Process. Mag..

[31] Chowdhary M.A.M. (2025). Financial Network Infrastructure: Scalability, Security and Optimization. Master’s Thesis.

[32] Lekidis A., Papageorgiou E.I. (2023). Edge-based short-term energy demand prediction. Energies.

[33] Li S., Yan Y. (2022). DATA-driven shock impact of COVID-19 on the market financial system. Inf. Process. Manag..

[34] Farzulla M. (2025). Infrastructure vs. Regulatory Shocks: Asymmetric Volatility Response in Cryptocurrency Markets. Res. Sq..

[35] Skålvik A.M., Saetre C., Frøysa K.E., Bjørk R.N., Tengberg A. (2023). Challenges, limitations, and measurement strategies to ensure data quality in deep-sea sensors. Front. Mar. Sci..

[36] Lu Z. (2022). Financial asset risk measurement based on smart sensor big data security analysis and bayesian posterior probability model. Wirel. Commun. Mob. Comput..

[37] Luo H., Liu Y., Zhang R., Wang J., Sun G., Niyato D., Yu H., Xiong Z., Wang X., Shen X. (2025). Toward edge general intelligence with multiple-large language model (Multi-LLM): Architecture, trust, and orchestration. IEEE Trans. Cogn. Commun. Netw..

[38] Wang M., Zhang Q., Liu X., Zhang J., Yu F., Zhang X., Zhao R. (2025). Research progress on multimodal data fusion in forest resource monitoring. Front. Plant Sci..

[39] Sun J. (2025). Research on Sentiment Analysis Based on Multi-source Data Fusion and Pre-trained Model Optimization in Quantitative Finance. SOcio-Econ. Stat. Res..

[40] Cao Y., Chen Z., Kumar P., Pei Q., Yu Y., Li H., Dimino F., Ausiello L., Subbalakshmi K., Ndiaye P.M. (2025). RiskLabs: Predicting financial risk using large language model based on multimodal and multi-sources data. Proceedings of the 2025 IEEE International Conference on Data Mining Workshops (ICDMW).

[41] Garcia D., Tolvanen J., Wagner A.K. (2022). Demand estimation using managerial responses to automated price recommendations. Manag. Sci..

[42] Lu J. (2025). Time-Series Foundation Models in Finance: Pretraining Corpora, Architectures, Financial Benchmarks, and Risk-Aware Evaluation. Proceedings of the 2025 2nd International Conference on Digital Economy and Computer Science.

[43] Rao H., Zhao Y., Hou X., Wang S., Wang H. (2025). Software engineering for large language models: Research status, challenges and the road ahead. arXiv.

[44] Han H., Lou Z., Wang Q., Xu L., Li Y. (2024). Introducing rich heterojunction surfaces to enhance the high-frequency electromagnetic attenuation response of flexible fiber-based wearable absorbers. Adv. Fiber Mater..

[45] Yang H., Lambotharan S., Derakhshani M., Hanzo L. (2026). LLM-enhanced space-air-ground-sea integrated networks. IEEE Communications Magazine.

[46] Cox D.R. (1958). The regression analysis of binary sequences. J. R. Stat. Soc. Ser. B Stat. Methodol..

[47] Chen T., Guestrin C. Xgboost: A scalable tree boosting system. Proceedings of the 22nd ACM Sigkdd International Conference on Knowledge Discovery and Data Mining.

[48] Ke G., Meng Q., Finley T., Wang T., Chen W., Ma W., Ye Q., Liu T.Y. (2017). Lightgbm: A highly efficient gradient boosting decision tree. Adv. Neural Inf. Process. Syst..

[49] Graves A. (2012). Long short-term memory. Supervised Sequence Labelling with Recurrent Neural Networks.

[50] Vaswani A., Shazeer N., Parmar N., Uszkoreit J., Jones L., Gomez A.N., Kaiser Ł., Polosukhin I. (2017). Attention is all you need. Adv. Neural Inf. Process. Syst..

[51] Zhou H., Zhang S., Peng J., Zhang S., Li J., Xiong H., Zhang W. Informer: Beyond efficient transformer for long sequence time-series forecasting. Proceedings of the AAAI Conference on Artificial Intelligence.

[52] Devlin J., Chang M.W., Lee K., Toutanova K. Bert: Pre-training of deep bidirectional transformers for language understanding. Proceedings of the 2019 Conference of the North American Chapter of the Association for Computational Linguistics: Human
Language Technologies, Volume 1 (Long and Short Papers).

[53] Shen Y., Zhang P.K. (2024). Financial sentiment analysis on news and reports using large language models and finbert. Proceedings of the 2024 IEEE 6th International Conference on Power, Intelligent Computing and Systems (ICPICS).

[54] Wu S., Irsoy O., Lu S., Dabravolski V., Dredze M., Gehrmann S., Kambadur P., Rosenberg D., Mann G. (2023). Bloomberggpt: A large language model for finance. arXiv.

